# Effectiveness of a theory-informed intervention to increase care home staff influenza vaccination rates: a cluster randomised controlled trial

**DOI:** 10.1093/pubmed/fdaf023

**Published:** 2025-03-30

**Authors:** David Wright, Jeanette Blacklock, Veronica Bion, Linda Birt, Allan Clark, Alys Wyn Griffiths, Cecile Guillard, Susan Stirling, Andy Jones, Richard Holland, Liz Jones, Thando Katangwe-Chigamba, Carys Seeley, Jennifer Pitcher, Helen Risebro, Sion Scott, Adam Wagner, Erika Sims, Saiqa Ahmed, Luke Cook, Amrish Patel

**Affiliations:** School of Healthcare, University of Leicester, University Road, Leicester LE1 7RH, UK; School of Healthcare, University of Leicester, University Road, Leicester LE1 7RH, UK; Norwich Clinical Trials Unit, University of East Anglia, Chancellors Drive, Norwich NR4 7TJ, UK; School of Healthcare, University of Leicester, University Road, Leicester LE1 7RH, UK; Norwich Medical School, University of East Anglia, Chancellors Drive, Norwich NR4 7TJ, UK; School of Medicine and Population Health, University of Sheffield, Beech Hill Road, Sheffield S10 2RX, UK; Norwich Clinical Trials Unit, University of East Anglia, Chancellors Drive, Norwich NR4 7TJ, UK; Norwich Clinical Trials Unit, University of East Anglia, Chancellors Drive, Norwich NR4 7TJ, UK; Norwich Medical School, University of East Anglia, Chancellors Drive, Norwich NR4 7TJ, UK; University of Exeter Medical School, Heavitree Road, Exeter EX12 2LU, UK; School of Medicine and Population Health, University of Sheffield, Beech Hill Road, Sheffield S10 2RX, UK; Norwich Clinical Trials Unit, University of East Anglia, Chancellors Drive, Norwich NR4 7TJ, UK; Norwich Clinical Trials Unit, University of East Anglia, Chancellors Drive, Norwich NR4 7TJ, UK; School of Economics, University of East Anglia, Chancellors Drive, Norwich NR4 7TJ, UK; Norwich Medical School, University of East Anglia, Chancellors Drive, Norwich NR4 7TJ, UK; School of Healthcare, University of Leicester, University Road, Leicester LE1 7RH, UK; Norwich Clinical Trials Unit, University of East Anglia, Chancellors Drive, Norwich NR4 7TJ, UK; National Institute of Health Research (NIHR) Applied Research Collaboration (ARC) East of England (EoE), Douglas House, 18 Trumpington Road, Cambridge CB2 8AH, UK; Norwich Clinical Trials Unit, University of East Anglia, Chancellors Drive, Norwich NR4 7TJ, UK; School of Medicine and Population Health, University of Sheffield, Beech Hill Road, Sheffield S10 2RX, UK; Askham Village Community, 13 Benwick Road, March PE15 0TX, UK; School of Economics, University of East Anglia, Chancellors Drive, Norwich NR4 7TJ, UK

**Keywords:** employees, influenza vaccination, long-term care facilities, nursing homes, residential homes, social care, staff

## Abstract

**Background:**

Care home staff’s (CHS’s) influenza vaccination rate in England is 30%–40%, below the 75% WHO recommendation. We describe the effectiveness of a theory-informed and feasibility-tested intervention (in-home clinics; posters/videos to address vaccination hesitancy and care home financial incentives for uptake) to improve CHS vaccination rates.

**Method:**

Recruited care homes in England with CHS vaccination rates <40% were randomised at the home level for intervention or control. Assuming a change in CHS vaccinated from 55% to 75%, 20% attrition, and 90% power, we required 39 homes per arm. Monthly data were collected throughout flu season. The difference in vaccination rates between the arms was compared using the intention-to-treat principle and a random effect logistic regression model.

**Findings:**

The mean % vaccination rate was 28.6% in control (*n* = 35) and 32.7% in intervention (*n* = 35) [odds ratio (OR) = 1.29, 95% confidence interval (CI): 0.68–0.4, *P* = .435]. In a sub-analysis, including only homes receiving at least one clinic, control was 28.6% (*n* = 35) and intervention was 41.7% (*n* = 23) (OR = 2.08, 95% CI: 0.67–2.70, *P* = .045).

**Interpretation:**

No effect on vaccination status was demonstrated. Within homes receiving clinics, a significant increase was observed. Process evaluation evidence suggests that starting 3 months into the influenza season partially explains this. Further evaluation initiating FluCare earlier is warranted.

## Research in context

### Evidence before this study

Previous interventions to increase care home staff (CHS) influenza vaccination have demonstrated modest increases in vaccination rates. This is frequently from very low baseline levels, if indeed a significant increase is identified at all. Researchers have identified causes of vaccine hesitancy in CHS including a lack of perceived need for vaccination or understanding of the benefits for both themselves and residents. Concerns regarding vaccine safety and a lack of prioritisation or support for staff vaccination by care home managers are also known barriers. The main barrier to seeking vaccination is, however, vaccine accessibility, with the provision of in-house vaccination clinics found to be critically important. Previous interventions have focused on only addressing a limited subset of known barriers to vaccination and have not engaged CHS or patient and public involvement members in their design.

### Added value of this study

This study provides evidence that an intervention, which is designed to address all known barriers, theory-informed, and developed with stakeholder engagement, significantly increases staff vaccination rates in homes where in-home flu vaccination clinics—the key intervention component—are delivered.

### Implications of available evidence

This research demonstrates that a theory-informed intervention may result in a larger effect than previously reported interventions. Effectiveness is dependent on the provision of in-house clinics within the care homes. Given the late delivery of the intervention in the trial, even greater impacts may accrue if delivered at the start of the flu season.

## Background

Influenza is a respiratory infection with a significant annual burden.^1^^,^^2^ Each year, there are approximately 1 billion infections, 3–5 million cases of severe illness, and up to 640 000 deaths globally.^3^^,^^4^ Older adults have a greater infection risk due to lower immunity caused by chronic comorbidities and immunosenescence, age-related immune system decline,^3^^,^^5^ leading to complications including acute respiratory symptoms, exacerbation of chronic conditions, and cardiovascular events.^6^ Approximately 70%–85% of influenza-related deaths and 50%–70% of influenza-related hospitalisations are among those over 65 years old.^7^

Influenza outbreaks in care homes contribute significantly to hospitalisations and mortality, with attack rates of 20%–40% during care home outbreaks^8^^,^^9^ and a median case-fatality rate of 6.5%.^10^

Vaccination is the most effective method of preventing influenza infections.^11^ Coverage rates in adults aged over 65 in England are 70%–80%;^12^ however, immunosenescence limits immune responses.^13–15^ Consequently, the WHO recommends at least 75% of CHS are vaccinated to protect residents.^16^ Vaccination of CHS is positively correlated with resident health outcomes,^17^^,^^18^ CHS health, and resident care quality.^19^^,^^20^

The UK Health Security Agency recommends that all CHS are vaccinated for influenza.^21^ The Health and Safety at Work Act (1974) and Health and Social Care Act (2008) emphasise that employers should assess and control occupational health risks, including facilitating staff influenza vaccinations. Despite this, CHS influenza vaccination rates are frequently less than 40%.^22–24^ To address this deficit, interventions to improve vaccination uptake in CHS are required.

Barriers to CHS influenza vaccination uptake have been identified as a lack of understanding and misconceptions of vaccine safety and effectiveness, fear of side effects, and inability to easily access the vaccine.^25^ The importance of support and leadership from management and organisations has also been reported as barriers and enablers to CHS vaccination uptake.^26–28^

A study in Hong Kong testing an intervention addressing vaccine safety and efficacy concerns reported a non-significant increase of 8% in the intervention arm.^29^ A ‘nudge’ intervention including a personalised letter from a high-profile figure raising professional responsibility to residents and the burden of influenza on colleagues similarly demonstrated an 8% increase in vaccination rate.^30^ However, the vaccination rate was only 28% in the intervention arm.^30^ A cross-sectional survey of CHS across 19 care homes found that, in decreasing importance, predictors for CHS vaccination were staff vaccination organised by the care home, awareness of regulations requiring vaccination, reminders to vaccinate, and information provision on vaccine need.^31^

**Figure 1:**
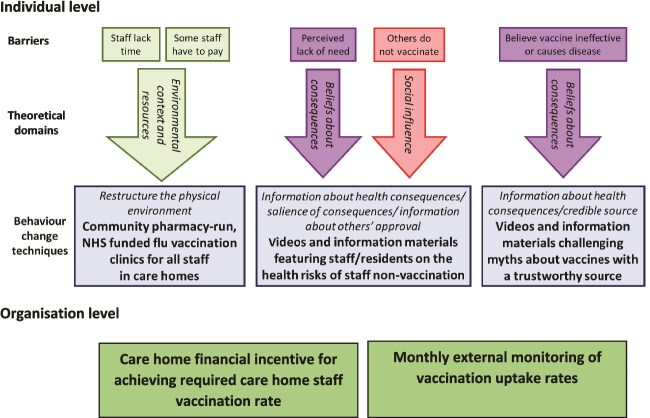
FluCare intervention. Adapted from^33^.

No CHS interventions to increase influenza vaccination rates have been designed or reported to simultaneously address all known barriers to uptake. Consequently, we developed an evidence-based, multi-level, theory-informed intervention to enhance CHS vaccination rates (Fig. 1). The design was not found to overlap with other clinical trials reported within the WHO International Registry.^32^

A feasibility study including the intervention, with process evaluation, demonstrated that a study was feasible, provided that a more effective approach for care home recruitment was identified and that CHS could be encouraged to watch the intervention videos.^33^ The aim of our study was therefore to assess the effectiveness of a theory-informed intervention,^34^ operationalised at both individual and organisational levels to improve CHS vaccination rates. The results of the process evaluation embedded in the trial and an economic evaluation will be published separately.

## Methods

### Trial design

A cluster randomised controlled, two-arm, open-label, effectiveness trial of FluCare, a behaviour change intervention designed to improve uptake of influenza vaccination by staff in care homes in England, compared to usual practice with monthly performance monitoring, was performed. The trial protocol, providing greater methodological detail, has been published,^35^ alongside the process evaluation protocol.^36^

### Study setting

Private, charity, corporate, local authority, or NHS care homes (both residential and nursing homes) in England that provide care for older adults.

### Recruitment

Expressions of interest for care home participation were initially sought between May and August 2022. Community pharmacies or medical practices willing to provide in-care-home vaccination clinics were identified by care homes and contacted by the research team.

Each year from September to March, all care homes in England are required to submit monthly flu vaccination rates for CHS and residents to the Department of Health and Social Care (DHSC) Capacity Tracker. Approval was given to use this to identify care homes in which <40% of staff received a flu vaccination during the 2021/2022 flu season. Care homes were emailed and mailed directly to seek expressions of interest. NIHR Clinical Research Networks, care home representative bodies (e.g. Care England), community pharmacy companies, social media campaigns, and care home networks were additionally used to promote the study and seek expressions of interest.

### Inclusion/exclusion criteria

Care homes expressing interest in participation were recruited according to the following inclusion criteria:

- Long stay for older residents or dementia registration
- Self-reported staff vaccination rate < 40%
- Signed up to, or willing to sign up to, the DHSC Capacity Tracker, and willing to provide weekly updates on the flu vaccine status of staff and residents

### Exclusion criteria

- Fewer than 10 staff members
- Participated in FluCare feasibility study^33^
- Participating in an existing trial of behaviour change interventions (NB: Staff allowed to concurrently participate in trials of coronavirus disease 2019 (COVID-19) treatments)

### Data collection measures

A site profile questionnaire (SPQ) was completed by each care home to confirm eligibility to participate prior to recruitment and randomisation. The SPQ included:

- Care home ownership [i.e. home type (private/charity/local authority/NHS owned)];
- Size (number of beds);
- Care home registration (i.e. residential/nursing/both);
- Staffing: number and type of staff and their working arrangements;
- Policies and procedures: infection control policies, relevant protocols/operating procedures, vaccine policy, guidance/education routinely provided;

At the end of follow-up, care home managers were requested to repeat the SPQ and confirm any changes that may have affected trial implementation during the trial period.

After randomisation, care homes were requested to keep a CHS log, which tracked the vaccination status of each staff member and staff sick days. This was submitted to the research team monthly.

Vaccination providers running in-home clinics kept a vaccination provider log with a list of all staff members vaccinated in a clinic. This was sent to the research team and the care home manager to update the CHS log and the DHSC Capacity Tracker.

Care home managers also kept a resident log, which recorded data on aggregate resident outcomes including the number of general practitioner (GP) consultations, planned hospitalisations, emergency hospitalisations, and mortality.

### Participation remuneration

All participating care homes received up to £500 for costs associated with facilitating research and data collection. Additional payments were made for process evaluation participation.

For each on-site clinic delivered, vaccination providers (GPs or community pharmacists) received a minimum payment of £300. For pharmacy-delivered clinics, this fee was partly comprised the NHS England flu administration fee, which was recouped for each vaccination administered. Vaccination providers received funding for delivering up to four clinics.

### Randomisation and masking

#### Sequence generation

The allocation sequence was generated electronically based on stratified randomisation with a by the percentage of staff identifying as non-white: <23% vs ≥ 23%. Blocked (random blocks of size 4 or 6) randomisation was used.

Classification of the percentage of staff identifying as non-white into either of the two stratification groups was determined based on SPQ data (completed prior to randomisation).

#### Allocation concealment mechanism

To ensure that the recruitment and care home-facing team could not bias the allocation to intervention or control, the team were concealed to the allocation sequence. Care homes were recruited, consented, and required to complete the baseline SPQ prior to allocation. The allocation process was managed by the Norwich Clinical Trials Unit (NCTU) data management team, who were not involved in recruitment or care home engagement.

#### Blinding

Due to the trial design, it was not possible to blind members of the research team (operational, management, statisticians, and health economic researchers).

#### Intervention and usual care

Supplementary file 1 provides the populated template for intervention description and replication checklist for the intervention.^37^ The multi-component intervention (Fig. 1) comprised:

- Online video of stakeholders describing the purpose and benefits of vaccination (GP, chief nurse, residents, and CHS) plus supporting information materials including posters and leaflets for displaying in each care home.
- GP and/or pharmacy vaccination provision comprising up to four vaccination clinics arranged through consultation between CHS and vaccination providers (VPs), organised around care home shifts.
- Monthly monitoring of CHS vaccination uptake rates (as in the control arm).
- Care home incentive scheme comprising £850 incentive if >70% of CHS received a flu vaccination, based on the CHS log.

Following allocation to intervention, care home managers were emailed the online video link to share with staff during team meetings and sent posters and leaflets promoting flu vaccination for distribution around the home. Community pharmacy and GP practice staff paired with the care home were requested to liaise with the care home manager, agreeing on suitable dates and times to deliver the in-home vaccination clinics. Clinics could be conducted alongside or separate from resident flu and/or COVID vaccination sessions. At the end of follow-up, care homes in which more than 70% of CHS received the flu vaccination received an £850 incentive payment.

Usual care included monthly and end-of-study data collection. Other than potential awareness of participation in the trial, no additional information was provided to staff. Outcome data were requested by the research team monthly and at the study end to assess face-value data quality, with feedback to the care home manager should issues be identified (e.g. highlighting missing observations). The feasibility study identified no reactivity bias from monthly data collection in isolation.^33^

Care home managers who discontinued protocol treatment were encouraged to remain in the trial for the purpose of providing follow-up information. All care homes that withdrew were included in the data analysis.

**Figure 2:**
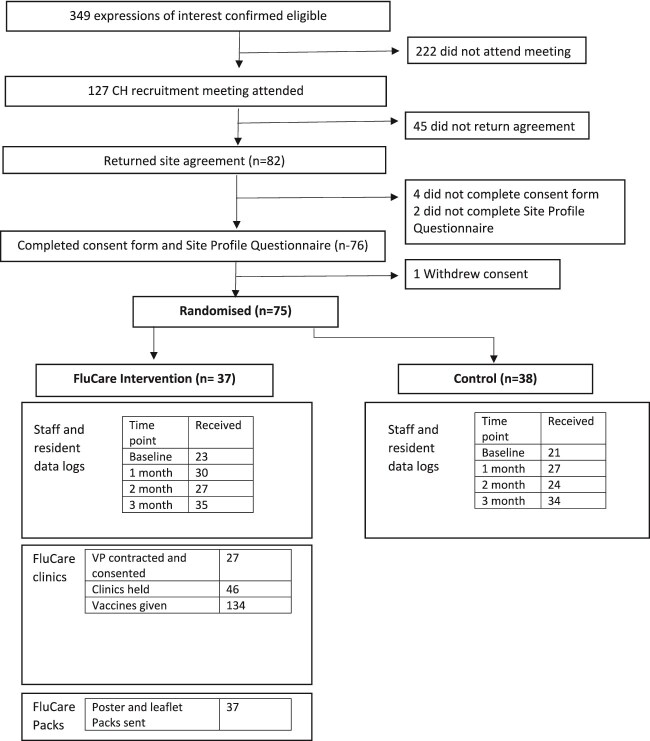
Consort diagram.

A summary of the recruitment and randomisation process is provided in Fig. 2.

#### Primary outcomes

The primary outcome measure was the total number of staff vaccinated in a flu season divided by the total number of staff employed at any point throughout that flu season (all directly contracted staff including care staff, cleaners, cooks, and administrative staff).

#### Secondary outcomes

- Staff flu vaccination rate at the end of November
- Number of staff sick days
- GP consultations concerning care home residents
- Care home resident hospitalisations
- Care home resident mortality

#### Sample size calculation

Based on the assumptions that the mean (SD) cluster size was 54 staff,^38^ a coefficient of variation of 0.48,^39^ a control vaccination rate of 55%, an intervention of 75%, intra-cluster correlation coefficient of 0.2, and 90% power, we required 39 care homes per arm at the two-tailed 5% level of significance (78 total), assuming 20% attrition. This sample size also provided over 90% power to detect a difference between a control rate of 40% and an intervention rate of 60%.

## Data analysis

### Statistical methods

Analysis was based on the intention-to-treat principle, using all available data. Vaccination rates were presented for each group separately and compared using a random effect logistic regression model at the staff level, with a random effect at the home. If staff data were missing, then the result sensitivity was assessed by imputation, assuming that those staff were not vaccinated. Secondary outcomes such as staff sickness absence and resident GP consultations, hospitalisations, and mortality were analysed at the level of the care home using linear regression. Assumptions were checked, and if violated, then either a nonparametric bootstrap or cluster-summary approach was used. The analysis considered first all staff, followed by the caregiver and non-caregiver staff groups separately. A key sub-group analysis was to compare outcomes in control homes to only intervention homes that received at least one vaccination clinic. Full details were agreed upon and documented in the statistical analysis plan (SAP) before the final analysis. The SAP was signed on 13 June 2023 and is available upon request to Dr. Amrish Patel (Amrish.patel@uea.ac.uk). STATA 17.0/SE (Texas) was used to conduct the statistical analysis. A health economics plan was also developed before the economic evaluation and is available upon request from Dr. Amrish Patel (Amrish.patel@uea.ac.uk).

### Process evaluation

Following Medical Research Council guidance,^40^ we conducted a mixed methods process evaluation,^36^ including quantitative and qualitative data collection to understand the implementation of the FluCare intervention and provide explanations for the observed effects in the cluster RCT. This is reported elsewhere.^41^

### Governance

The University of East Anglia was the trial sponsor, with responsibility for the overall management of the FluCare trial delegated to the co-chief investigators (A.P. and D.W.) and NCTU. Ethical approval for the trial was obtained from the University of East Anglia, Faculty of Medicine and Health Ethics Committee on 1 August 2022 (study approval number ETH2122-2419), and governance approval was received from the UK Health Research Authority on 15 August 2022 (study approval number IRAS 316820).

### Role of funding source

NIHR reviewers provided comments on the study design at various points, approved protocols and amendments thereof, and decided to submit the manuscript. They were not involved in any other elements.

## Results

A total of 75 homes were randomised (37 intervention and 38 control). Table 1 provides a summary of baseline characteristics for both arms. There were slightly more homes with nursing care-only status and charity ownership in the control arm. Control arm homes cared for slightly more residents on average but employed more staff, and they were more likely to be in permanent roles. Intervention arm clinics were held between November 2022 and April 2023 (with 80% of the clinics taking place from February 2023 onwards).

**Table 1:** Baseline characteristics.

	Control (*n* = 38)	Intervention (*n* = 37)
Care home registration		
Residential (*n* (%))	13 (34.2%)	21 (56.8%)
Nursing (*n* (%))	7 (18.4%)	0
Both residential & nursing (*n* (%))	18 (47.4%)	16 (43.2%)
Care home ownership		
Local authority (*n* (%))	0	1 (2.7%)
Charity (*n* (%))	9 (23.7%)	2 (5.4%)
Privately owned (*n* (%))	29 (76.3%)	34 (91.9%)
Care home residents		
Mean (SD) number of residents per home	42.7 (21.4)	39.4 (16.7)
Median (IQR) number of residents per home	40 (25–52)	39 (27–54)
Care home staff (per care home) in SPQ log		
Permanent staff mean (SD), median (IQR)	60.2 (35.8) 55.5 (39–64)	48.6 (19.5) 46 (33–62)
Bank staff mean (SD), median (IQR)	5.2 (6.7) 2(1–7)	3.7 (4.0) 3 (0–5)
Agency staff mean (SD), median (IQR)	4.5 (4.3) 3.5 (2–6)	2.9 (3.1) 2 (0–5)
Voluntary staff mean (SD), median (IQR)	2.6 (8.8) 0 (0–1)	1.1 (1.6) 1.2 (0–2)
How do your staff receive their flu vaccinations?		
In the care home by GP, *N*(%)	9 (23.7%)	11 (29.7%)
In the care home by pharmacist, *N*(%)	7 (18.4%)	6 (16.2%)
In GP practice, *N*(%)	32 (84.2%)	33 (89.2%)
In community pharmacy, *N*(%)	32 (84.2%)	28 (75.7%)
Don’t know, *N*(%)	3 (7.9%)	5 (13.5%)
Any incentives for CHS to get the flu vaccine?	0	2 (5.4%)
Any protocols in place for when staff are sick with the flu or other infections?	33 (86.8%)	31 (83.8%)
System to collect and record information on staff flu vaccination status?	27 (71.1%)	26 (70.3%)
Staff ethnicity (mean [SD] per care home)		
White/White British, mean (SD)	37.2 (39.9)	32.5 (18.7)
Black African/Caribbean/Black British, mean (SD)	8.0 (11.0)	4.2 (6.4)
Mixed/multiple ethnic group, mean (SD)	2.3 (5.7)	3.8 (7.1)
Asian/Asian British, mean (SD)	9.5 (14.3)	5.7 (10.4)
Other ethnic group, mean (SD)	0.6 (1.5)	0.4 (1.3)
Staff gender (mean [SD] per care home)		
Man, mean (SD)	11.7 (13.1)	8.8 (7.6)
Woman, mean (SD)	48.2 (36.1)	43.1 (17.9)
Other, mean (SD)	0.1 (0.5)	0.1 (0.2)
Unknown, mean (SD)	4.6 (19.3)	0.1 (0.3)

The results of the analysis of primary and secondary outcomes are presented in Table 2. No difference was shown in our intention-to-treat analysis of the primary outcome measure (when all intervention care homes were included) in the analysis, with intervention 32.7% versus usual care 28.6% [OR: 1.29 (95%: CI 0.68, 2.44); *P* = .435]. We additionally adjusted this for care home type, and the results were similar [OR: 1.35 (95% CI: 0.67, 2.70); *P* = .404]. However, restricting the intervention group to only those homes that held at least one vaccination clinic led to a difference of 13.1% in vaccination between arms, which was statistically significant, a difference in favour of the intervention [intervention: 41.7%; control: 28.6%; OR 2.08; (95% CI 1.03, 4.21); *P* = .042]. Further adjustment for care home type again gave similar results [OR 2.35 (95% CI 1.02, 5.39); *P* = .044]. Additional analysis assuming staff with missing vaccination status are non-vaccinated is presented in Appendix Table A1. This has little impact on the interpretation of the results.

**Table 2:** Primary and secondary outcome measure comparisons.

Outcome Measure	Control	Intervention	Odds ratio (95% CI) minimally adjusted^d^	ICC	*P*
Vaccination rate^a^ % vaccinated/care home	(*n* = 35) 28.6%	(*n* = 35) 32.7%	1.29 (0.68, 2.44)^d^	0.34	.435
Sensitivity analysis^b^			1.35 (0.67, 2.70)	0.32	.404
Vaccination rate in homes receiving a vaccine clinic % vaccinated/care home	(*n* = 35) 28.6%	(*n* = 23) 41.7%	2.08 (1.03, 4.21)^d^	0.33	.042
Sensitivity analysis^b^			2.35 (1.02, 5.39)	0.32	.044
Staff sickness absence rate Mean (SD) days per staff member per year	(*n* = 32) 7.21 (5.28)	(*n* = 35) 7.35 (5.83)	0.14 (−2.61, 2.88)^d^		.920
Residents’ episodes of GP consultations^c^ Mean (SD) rate per resident per year	(*n* = 32) 10.14 (8.59)	(*n* = 35) 9.64 (14.04)	−0.49 (−6.27, 5.30)^d^		.867
Hospitalisations: planned and emergency Mean (SD) rate per 100 residents per year	(*n* = 32) 55.93 (40.19)	(*n* = 35) 76.62 (80.62)	20.61 (−11.18, 52.41)^d^		.200
Hospitalisations: emergency only Mean (SD) rate per 100 residents per year	(*n* = 32) 45.78 (38.51)	(*n* = 35) 62.52 (63.48)	16.50 (−9.45, 42.45)^d^		.209
Hospitalisations: planned only Mean (SD) rate per 100 residents per year	(*n* = 32) 10.15 (17.35)	(*n* = 35) 14.10 (32.92)	4.12 (−8.84, 17.07)^d^		.528
Resident mortality Mean (SD) rate per 100 residents per year	(*n* = 32) 57.83 (38.12)	(*n* = 35) 49.88 (35.41)	−7.56 (−24.97, 9.84)^d^		.389

a Excluding missing vaccination rate.

b Analysis additionally stratified by care home type (residential/nursing/both) and care home ownership (LA/charity/private).

c In-person consultations, virtual consultations and phone calls.

d Only for stratification variables: % of staff identified in staff as non-white: <23% vs ≥23%.

Some care homes reported vaccination status for voluntary staff, which were not included in the SAP. Including these made no difference to the outcomes. The percentage vaccinated per care home including voluntary staff was 28.7% in the control arm and 32.6% in the intervention arm, *P* = .460. Within only care homes with vaccination clinics, it was 28.7% and 41.7%, respectively, *P* = .045.

No significant differences in secondary outcome measures between treatments were observed.

## Discussion

### Main finding of this study

When considering the primary outcome measure and using an intention-to-treat analysis, no statistically significant difference in CHS vaccination rate was detected despite our theory-informed intervention. When including only homes that organised one or more in-house clinic, a 13% increase in CHS vaccination status was detected. Given the low baseline and target vaccination rate of 75%, this is, however, a relatively small increase.

### What is already known on this topic

The burden of influenza in older adults is significant.^1–3^ Vaccination is the most effective method of protecting against influenza, thus the WHO recommends that at least 75% of those caring for older adults are vaccinated.^16^ Despite this, <40% of CHS are vaccinated for influenza.^22–24^

Previous interventions to increase CHS influenza vaccination found modest increases in vaccination rates, if at all. Known barriers to vaccine uptake in CHS include a lack of perceived need, vaccine safety concerns, and a lack of prioritisation/support by managers. The main barrier is, however, vaccine accessibility, with in-house vaccination clinics critically important. Previous interventions have addressed a subset of known barriers and have not engaged CHS or patient and public involvement members in their design.

### What this study adds

The study evaluates the effectiveness of an intervention aiming to increase CHS vaccination rates, addressing all known barriers. The study was well-conducted, following all current standards for the delivery of cluster randomised controlled trials, and recruited almost to the sample size. Furthermore, it followed Medical Research Council guidance on the development and evaluation of complex interventions,^40^ using theory to inform intervention development, a logic model to identify outcome measures, and a feasibility study to confirm trial design appropriateness.

Feedback from the embedded process evaluation suggests that the lack of a statistically significant difference in the intention-to-treat result may be explained by the intervention starting in most cases at least 3 months into influenza season. Fewer clinics and vaccinations are expected given the shorter time period available and the lower perceived need.

The intervention should not be simplified to just in-house clinics, as emerging results from the process evaluation indicate some impact from the behaviour change information. Our feasibility study^33^ suggests that the posters should have been effective at dispelling myths regarding vaccination safety/effectiveness, whilst reinforcing the health benefits. However, our process evaluation for the current study identified variations in implementation and engagement; not all intervention care homes implemented all intervention components. This could have also contributed to the low vaccine uptake. For example, although videos were used, most staff did not view them; hence, their additional contribution is unclear. An economic evaluation of the intervention comparing the costs and outcomes is to follow.

Financial incentives were also introduced to encourage care home managers, but again, the additional benefit is unclear. The process evaluation within our feasibility study suggested that manager leadership would be pivotal, and without this, clinics may not occur. Our process evaluation should capture this.^36^

The results do however suggest that an intervention delivered from the beginning of influenza season is worth testing further to establish whether an extended time period, with organised in-house clinics, has a greater effect.

## Limitations of this study

Neither the research team nor the care homes were blinded to allocation, and therefore reporting bias is possible, particularly if we had just relied on care home reports on vaccination status. The collation of data from the in-house clinics enabled us to triangulate results in the intervention arm, providing more confidence. The relatively low vaccination rate reported in the control arm suggests that reactivity bias was limited.

Our initial recruitment methods (via care home leadership and representative groups) were not very effective. Individually emailing all eligible care homes in England to seek expressions of interest meant we recruited to sample size, but most homes were recruited 3 or 4 months into the season, limiting opportunities to provide vaccinations and participation incentives.

Our feasibility study^33^ highlighted the significant reporting burden on care home managers during influenza season. To not jeopardise the quality and completeness of primary outcome data and other critical variables, some relevant contextual variables (e.g. influenza outbreaks) were not requested. Such context is captured in the embedded process evaluation.

In this cluster randomised controlled trial of a multi-component intervention to increase flu-vaccination uptake by CHS, we found no statistically significant effect, though limiting our analysis to homes receiving clinics did imply vaccinations could be increased. As a secondary analysis, our interpretation should be cautious, and delivery was often late in the flu season. Our recommendation is that the intervention is tested again with delivery before flu season as the intervention shows potential.

## Supplementary Material

FluCare_StatisticalAnalysisPlan_fdaf023

FluCare_TIDieR_checklist_fdaf023

Protocol_FluCare_Phase_3_v1_4_27Feb2023_fully_signed_fdaf023

## Data Availability

The FluCare data and materials are available for secondary research purposes. In the first instance, requests should be directed to Dr Amrish Patel (amrish.patel@uea.ac.uk). Release of data may be subject to completion of a data-sharing agreement.

## Appendix

**Table A1:** Primary outcome (including missing: assumed non-vaccinated).

Outcome Measure	Control	Intervention	Odds ratio (95% CI) minimally adjusted^c^	ICC	*P*
Vaccination rate^a^ % vaccinated/care home	(*n* = 35) 24.2%	(*n* = 35) 26.5%	1.17 (0.66, 2.06)^c^	0.28	.592
Sensitivity analysis^b^			1.22 (0.66, 2.23)	0.26	.529
Vaccination rate in homes receiving a vaccine clinic % vaccinated/care home	(*n* = 35) 24.1%	(*n* = 23) 35.4%	1.81 (0.99, 3.32)^c^	0.26	.054
Sensitivity analysis^b^			1.89 (0.94, 3.82)	0.25	.075

a Missing vaccination status assumed to be non-vaccinated.

b Analysis additionally stratified by care home type (residential/nursing/both) and care home ownership (LA/charity/private).

c Only for stratification variables: % of staff identified in staff as non-white: <23% vs ≥23%.

## Trial status

The current protocol version number is version 1.4 (27 February 2023). Recruitment began on 1 September 2022 and was completed by the end of 30 June 2023.

## Notes

**David Wright**, Professor of Health Services Research

**Jeanette Blacklock**, Senior Research Associate

**Veronica Bion**, Clinical Trial Manager

**Linda Birt**, Associate Professor of Palliative Care and Frailty

**Allan Clark**, Associate Professor of Medical Statistics

**Alys Wyn Griffiths**, Senior Research Fellow

**Cecile Guillard**, Database Programmer

**Susan Stirling**, Statistician

**Andy Jones**, Professor of Public Health

**Richard Holland**, Professor of Public Health

**Liz Jones**, Public and Patient Involvement

**Thando Katangwe-Chigamba**, Senior Research Associate

**Carys Seeley**, Senior Research Associate

**Jennifer Pitcher**, Senior Research Associate

**Helen Risebro**, Senior Research Associate, Health Economics

**Sion Scott**, Associate Professor of Behavioural Medicine

**Adam Wagner**, Associate Professor of Health Economics

**Erika Sims**, Research Lead

**Saiqa Ahmed**, Public and Patient Involvement

**Luke Cook**, Registered Care Home Manager

**Amrish Patel**, Associate Professor of Economics
