# Supplementary material for: Effectiveness of a theory-informed intervention to increase care home staff influenza vaccination rates: a cluster randomised controlled trial
Source: J Public Health (Oxf). 2025 Mar 30;47(2):246–57. doi: 10.1093/pubmed/fdaf023 (PMC12123319; doi:10.1093/pubmed/fdaf023)
Supplement: FluCare_StatisticalAnalysisPlan_fdaf023 [file flucare_statisticalanalysisplan_fdaf023.pdf]

FluCare Phase 3

FluCare Phase 3: Estimating the effectiveness and cost-effectiveness of a complex intervention to increase care home staff influenza vaccination rates

Statistical Analysis Plan (SAP)

Version 1.0

12th June 2023

Name	Title	Signature	Date
Amrish Patel	Co-Chief Investigator		12/06/23
David Wright	Co-Chief Investigator		13/06/23
Allan Clark	Statistician		12/06/23
Erika Sims	Research Lead	Erika Sims	12/06/2023
Veronica Bion	Trial Manager		12/06/2023

SAP REVISION HISTORY

Document Name	Version No.	Reason for Revision	Effective Date

1.0 Administrative Information

Sponsor:	University of East Anglia
Sponsor Reference:	22_PR_0968 - R209939
Funder:	National Institute for Health and Care Research – Public Health Research
Funder Reference:	NIHR133455
Trial Registration:	ISRCTN 22729870
Trial Identifier:	
CTA:	
NRES:	
IRAS:	316820
Chief Investigator:	Dr Amrish Patel / Professor David Wright
Trial Statistician:	Allan Clark
UKCRC Trials Unit:	NCTU
Latest Protocol:	1.4

2.0 Introduction

2.1 Background and Rationale
This is provided in section 5.1 of the protocol

2.2 Objectives
The overall trial objectives are provided in section 5.2, however this SAP covers the following:

1. Estimate the effect of the intervention on staff vaccination rates (primary outcome) and secondary outcomes identified in the logic model (eg residents' morbidity and mortality)

(the second objective listed in the protocol will be considered in a separate HEAP)

3. Relate variations in intervention fidelity to intervention effectiveness (in an embedded process evaluation):

3. To explore the effects of individual intervention components on the primary outcomes.

3.0 Study Methods

3.1 Trial Design
A two-arm open label definitive effectiveness trial of FluCare

Intervention: a behaviour change intervention designed to improve uptake of influenza vaccination by staff in care homes in England. The intervention comprises the following: online videos of stake holders (GP, nurse, residents and care home staff) and supporting information materials including posters and leaflets; care home incentive scheme comprising incentive payment if proportion of staff receiving a flu vaccination exceeds a specified limit; GP and/or community pharmacy vaccination provision comprising up to 4 vaccination clinics.

Control (Usual Care): although the care home manager will be aware that the care home is participating in the trial, no additional information will be provided to staff.

3.2 Allocation
The sequence will be generated using REDCAP based on stratified randomisation with a binary variable of the percentage of staff identifying as non-white. Blocked randomisation will be undertaken, however given the small number of homes randomised in order to ensure that the risk of imbalance is small a small block size will be used and specified in the allocation system specification.

Stratification is by percentage of staff identifying as non-white following consent of care home (< 23% vs 23% or more), information collected on the site profile questionnaire prior to randomisation, and whether or not care home has a paired vaccination provider or not,

3.3 Sample Size

This is provided in section 6.7 of the protocol but is repeated below.

Based on the assumptions that mean (sd) cluster size is 54 staff (25), a coefficient of variation of 0.48 (based on a recent study [61]), control vaccination rate is 55% (assumed higher than the historical rate as COVID has increased interest in vaccination), intervention 75%, intra-cluster correlation coefficient of 0.2 and with 90% power, we require 31 care homes per arm at the two tailed 5% level of significance (62 total). This would also provide 80% power to detect the same difference in the caregiving (non-caregiving) staff subgroup, assumed 40 per care home (14 per care home). We recruit an additional 8 homes per arm to allow for 20% attrition making the final intended sample size of 78 homes in total. The effect of COVID on the control vaccination rate is uncertain. Our sample size also provides over 90% power to detect a difference between a control rate of 40% and intervention rate of 60%. This would still represent a 50% relative increase in vaccination rates than these homes have achieved historically. Such an increase has meaningful benefits for residents since evidence suggests a linear relationship between staff vaccination coverage and resident health [13, 14].[10][11].

3.4 Framework

The FluCare trial is to determine whether vaccination rates are increased following the implementation of the intervention and therefore is testing for superiority. Secondary outcomes will also be tested for superiority.

3.5 Timing of outcome assessments

The schedule of outcome assessments is given in section 6.6 of the protocol and is repeated below

	**Care Home Manager and Staff timelines**								
	Enrolment	Allocation	Post-allocation (months)						Post-intervention
TIMEPOINT**	$-t_1$	0	1	2	3	4	5	6	
ENROLMENT:									
Eligibility screen	X								
Informed consent	X								
Allocation		X							
Training: Excel Spreadsheet	X	X							
INTERVENTIONS:									
Arm A (usual care)			←	———	———	———	———	→	

4

Arm B (behaviour change intervention)			←					→	
ASSESSMENTS:									
Care Home profile questionnaire	X							X	
Data spreadsheet completion	X		X	X	X	X	X	X	
Mechanisms of action questionnaire (+ consent)*	X								X
Understanding Decision Making questionnaire (+consent)**	X								X
Interviews (Managers and staff)*									X

Flu vaccination providers (Pharmacist / GP practice) timelines (Arm B)									
ENROLMENT:									
Eligibility screen	X								
Informed consent	X								
Training: Vaccination Log	X								
ASSESSMENTS:									
Vaccination log completion and send to NCTU			Up to 4 vaccination clinics#						
Interviews*									X

3.6 Interim analyses and stopping guidance
There are no interim analyses planned for FluCare

3.7 Timing of analyses
The analysis will be done once the database is locked and the SAP approved once all of the outcome data has been collected.

4.0 Statistical Principles

4.1 Levels of statistical significance
A 5% level of significance and 95% confidence intervals will be used throughout.

4.2 Treatment dose

Compliance with the intervention will be reported as per the table below.

	Intervention delivery
	(N=)
No of intervention arm care homes reporting one or more flu vaccination clinics based on vaccination logs.	
No of intervention care homes with at least one member of staff playing video on one or more occasion	
No of intervention care homes displaying one or more poster?	

4.3 Protocol deviations

Protocol deviations will be discussed at the TMG and will be reported as a list. A decision will be made, regarding whether the care home should be excluded from the analysis.

4.4 Analysis populations

Analyses will be undertaken on an Intention to Treat basis in which care homes (and corresponding participating staff members) will be analysed in the group to which they were allocated regardless of their compliance with the intervention). If it is reported that staff are vaccinated prior to leaving a care home their data will be included within the analysis; if the data is missing or they are not vaccinated then it will be treated as missing data. The consort diagram will include the number of care homes who were randomised in error, if any – for example those randomised but which did not fulfil all the eligibility criteria.

5.0 Trial Population

5.1 Screening data

The following data and tables will be presented for all screened care homes.

Table 5.1.1 Screening data: number of care homes returning Expression of Interest (EOI)

Number of care homes returning full completed EOI	Number eligible	Number consenting	Number randomised

Eligibility

Care homes - Inclusion criteria:

- Long stay for older residents or dementia registration
- self-reported staff vaccination rate <40%
- signed up to, or willing to sign up to the DHSC Capacity Tracker and willing to provide weekly updates on flu vaccine status of staff and residents

Care homes - Exclusion criteria

- Fewer than 10 staff members
- Participated in FluCare feasibility study

Care home Staff

- All staff working at the care home for questionnaire completion only

Care home Residents

- All residents irrespective of whether they are permanent or respite residents for aggregate data

Eligibility of care homes will be reported as below

Table: Reason for Care home Eligibility

	Frequency: N (%)
Pre-screening eligibility criteria met	
**Reason for exclusion**	
a) Absence of inclusion criteria	
Care home registered for people aged over 65 years or people living with dementia	
Self-reported staff vaccination rate < 40%	
Signed up to, or willing to sign up to, the DHSC Capacity Tracker for uploading flu numbers (or care home group head office does so on behalf of care home)	
b) Presence of exclusion criteria	
Fewer than 10 staff members	

5.3 Recruitment and participant flow

The Consort diagram will be used to summarise the following information:

Number of care homes assessed for eligibility
- Number of care homes eligible at screening
- Number of care homes ineligible at screening

Number of care homes recruited
Number of care home staff at baseline
Number of care homes randomised to each trial arm

Number of care homes lost to follow-up
Number of care home staff lost to follow-up
Number of care homes discontinuing the intervention
Number of care homes withdrawing from the intervention
Number of care homes withdrawing from the trial
Number of care home staff included in the primary analysis

5.4 Withdrawal information

Reason for, and timing of, withdrawal of consent of care homes and care home staff will be indicated in the Consort diagram

5.5 Baseline Care home characteristics

	REDCap variable	Control	Intervention
**Care home registration:**	spq4_registration		
Residential (n (%))			
Nursing (n (%))			
Both residential & nursing (n (%))			
**Care home ownership:**	spq2_ownership		
Local authority (n (%))			
Charity (n (%))			
Privately owned (n (%))			
**Care home residents**	spq5_residents		
Total			
Mean (SD) number of residents per home			
Median (IQR) number of residents per home			
**Care home staff (per care home) in SPQ log**			
Permanent staff mean (SD), median (IQR)	spq7_total_ps		
Bank staff mean (SD), median (IQR)	spq7_total_bk		

Agency staff mean (SD), median (IQR)	spq8_tt_ag		
Voluntary staff mean (SD), median (IQR)	spq8_tt_vteer		
**Staff role (comprising sum of permanent staff, bank staff, agency staff and volunteers in each role) (per care home)**			
Management<br>mean (SD), median (IQR)	spq7_management_ps<br>spq7_management_bk<br>spq8_management_ag<br>spq8_management_vteer		
Admin<br>mean (SD), median (IQR)	spq7_admin_ps<br>spq7_admin_bk<br>spq8_admin_ag<br>spq8_admin_vteer		
Direct care team (categorised as "registered nurse" or "other" for permanent staff & bank staff; no equivalent categorisation for agency staff or volunteers, so all summed together into single "direct care" category)<br>mean (SD), median (IQR)	spq7_directcare_oth_ps<br>spq7_directcare_oth_bk<br>spq7_directcare_rn_ps<br>spq7_directcare_rn_bk<br>spq8_directcare_ag<br>spq8_directcare_vteer		
Cleaning staff<br>mean (SD), median (IQR)	spq7_cleaning_ps<br>spq7_cleaning_bk<br>spq8_cleaning_ag<br>spq8_cleaning_vteer		
Kitchen staff<br>mean (SD), median (IQR)	spq7_kitchen_ps<br>spq7_kitchen_bk<br>spq8_kitchen_ag<br>spq8_kitchen_vteer		
Activities co-ordinator<br>mean (SD), median (IQR)	spq7_activities_ps<br>spq7_activities_bk<br>spq8_activities_ag<br>spq8_activities_vteer		
Maintenance<br>mean (SD), median (IQR)	spq7_maintenance_ps<br>spq7_maintenance_bk<br>spq8_maintenance_ag<br>spq8_maintenance_vteer		
Mixed roles<br>mean (SD), median (IQR)	spq7_mixed_ps<br>spq7_mixed_bk<br>spq8_mixed_ag<br>spq8_mixed_vteer		
Other<br>mean (SD), median (IQR)	spq7_other_ps<br>spq7_other_bk<br>spq8_other_ag		

	spq8_other_vteer		
**Total staff per home from SPQ log** mean (SD), median (IQR)			
**Full / part time staff**			
Full time (total N)	spq10_fulltime		
Part time (total N)	spq10_parttime		
**Shift Pattern Availability**			
12 hour shifts N (% of all care homes)	spq11_shiftpatterns___1		
6 hour shifts N (% of all care homes)	spq11_shiftpatterns___2		
Weekday only N (% of all care homes)	spq11_shiftpatterns___3		
Weekend only N (% of all care homes)	spq11_shiftpatterns___4		
Day shifts only N (% of all care homes)	spq11_shiftpatterns___5		
Night shifts only N (% of all care homes)	spq11_shiftpatterns___6		
Other N (% of all care homes)	spq11_shiftpatterns___7		
**Ethnicity**			
White / White British N (%)	spq12_white		
Black African/Caribbean/Black British N (%)	spq12_black		
Mixed/multiple ethnic group N (%)	spq12_mixed		
Asian / Asian British N (%)	spq12_asian		
Other Ethnic group N (%)	spq12_other		
Ethnic group unknown N (%)	spq12_unknown		
**Staff gender**			
Man N (%)	spq13_man		
Woman N (%)	spq13_woman		
Other N (%)	spq13_other		
Unknown N (%)	spq13_unknown		
**How do your staff receive their flu vaccinations?**			
In the care home by GP N(%)	spq14_vax_how___1		
In the care home by pharmacist N(%)	spq14_vax_how___2		
In GP practice N(%)	spq14_vax_how___3		
In community pharmacy N(%)	spq14_vax_how___4		
Other N(%)	spq14_vax_how___5		
Don't know N(%)	spq14_vax_how___6		
**Methods used to communicate information about flu vaccination**			
Mandatory training N (%)	spq15_comms_fluvac___1		
Meetings N (%)	spq15_comms_fluvac___2		
Posters N (%)	spq15_comms_fluvac___3		
Videos N(%)	spq15_comms_fluvac___4		
Leaflets N(%)	spq15_comms_fluvac___5		
Emails N(%)	spq15_comms_fluvac___6		

Other N(%)	spq15_comms_fluvac___7		
None	spq15_comms_fluvac___8		
**Methods used by management to communicate general information directly to staff**			
Email N(%)	spq16_comms_ginfo___1		
WhatsApp/ Messaging App N(%)	spq16_comms_ginfo___2		
Online platform N(%)	spq16_comms_ginfo___3		
Text N(%)	spq16_comms_ginfo___4		
Phone call N(%)	spq16_comms_ginfo___5		
Paper handouts N(%)	spq16_comms_ginfo___6		
Meetings N(%)	spq16_comms_ginfo___7		
Other N(%)	spq16_comms_ginfo___8		
**Incentives for care home staff to get the flu vaccine?**	spq17_fluvac_incent		
Yes N(%)			
No N(%)			
**Methods used to inform staff about infection control policies or protocol changes in the care home**			
Email N(%)	spq18_comms_cp___1		
WhatsApp/ Messaging App N(%)	spq18_comms_cp___2		
Online platform N(%)	spq18_comms_cp___3		
Text N(%)	spq18_comms_cp___4		
Phone call N(%)	spq18_comms_cp___5		
Paper handouts N(%)	spq18_comms_cp___6		
Meetings N(%)	spq18_comms_cp___7		
Other N(%)	spq18_comms_cp___8		
**Which vaccine (if any) does care home have policy in place for?**	spq19_vac_policies		
**Any protocols in place for when staff are sick with the flu or other infections?**	spq20_sick_protocols		
Yes N(%)			
No N(%)			
**System to collect and record information on staff flu vaccination status?**	spq21_fluvac_data		
Yes N(%)			
No N(%)			
**Any protocol or guidance change with respect to staff being personally vaccinated against flu during the last year? (yes/no)**	spq22_protocol_chg		

Yes N(%)			
No N(%)			
**Aware of any upcoming changes/events that might impact delivery of FluCare within your team?**	spq23_upcoming_chg		
Yes N(%)			
No N(%)			

6.0 Analysis

6.1 Outcome definitions

6.1.1 Primary Outcome

The primary outcome will be staff flu vaccination rate and will be calculated as the total number of staff vaccinated in a flu season divided by the total number of staff employed at any point throughout that flu season (all directly contracted staff (care staff, cleaners, cooks, administrative staff) + agency staff).

6.1.2 Secondary Outcomes

The secondary outcomes are
1. Staff flu vaccination rate disaggregated by care-giving and non-care giving roles;

3. Number of staff sick days captured as the total number per care home;

4. Residents' episodes of GP visits captured as the total number per care home;

5. Residents' episodes of hospitalisation captured as the total number per care home;

6. Residents' mortality captured as the total number per care home.

6.1.3 Process Evaluation outcomes

The primary aim of this analysis will be to summarise the amount (or dose) of the intervention received in each intervention home. The outcomes will be summarized using tabulations and descriptive statistics of process items including: the number of posters in each care home, the number of times a video is viewed and, the average length of time watching the training videos.

a) The association between the proportion of vaccinated in each home and the process items listed above will be assessed at the home level in the intervention arm by estimating the correlation coefficient.
b) Each home will be classified using the process items listed above and a test-for-trend will be undertaken, including the control homes with zero dose, to assess if the amount of the intervention received is associated with the outcome.

Due to lower than anticipated MAQ response uptake we have scaled back the analysis in the protocol.

We will compare the change in each MAQ question between the control and intervention arms using a linear mixed model. The average for each MAQ question per care home will be correlated with the care-home vaccination rates.

6.2 Analysis Methods

6.2.1 Primary outcome

Analysis of the primary outcome will be based on the intention-to-treat principle, using all available data. Vaccination rates will be presented for each group separately, and the difference between them obtained and compared using a random effect logistic regression model at the staff-level (Stata command: xtlogit). The random effect will be the care home. If staff data are missing, then the results' sensitivity will be assessed by assuming that missing data are not vaccinated. The primary analysis will remain the observed data analysis, but it is worth noting that the default status on the vaccination log is 'not vaccinated' unless changed.

Table:

	Summary statistics		Minimally adjusted (only for stratification variables)		Fully adjusted (if required)	
Outcome	Control (n=)	Intervention (n=)	Odds Ratio (95% CI)	p-value	Odds Ratio (95% CI)	p-value
Vaccination rate						

6.2.2 Secondary outcomes

The analysis will consider firstly, all staff, then all care giver and non-care giver staff groups separately these analyses will be undertaken using a random effect logistic regression model separately for care giver and non-care givers.

The remaining secondary outcomes are measured at a care-home level and will be analysed without random effects model but at an aggregate level. The modelling details for each outcome are given below:

Outcome	Staff flu vaccination rate disaggregated by care-giving and non-care giving roles
Effect size	Odds ratio
Primary Analysis model	Random effect logistic regression
Sensitivity analysis	None
Missing data	Assumed to be unvaccinated.
Other comments	

Outcome	Total Number of staff sick days per care home
Effect size	Mean difference
Primary Analysis model	Linear regression model of the summary statistics for each care home, the intervention group will be a fixed effect along with the number of staff at the care home.
Sensitivity analysis	None
Missing data	Care homes with missing data will be excluded from the analysis.
Other comments	If the distribution is not normal, but rather Poisson or negative binomial then that distribution will be used. This will be decided by selecting the best fitting model (i.e. that with the highest log-likelihood will be used)

Outcome	Residents' episodes of GP visits
Effect size	Mean Total number of GP visits per care home
Primary Analysis model	Linear regression model of the summary statistics for each care home, the intervention group will be a fixed effect along with the number of residents at baseline
Sensitivity analysis	None
Missing data	Care homes with missing data will be excluded from the analysis.
Other comments	If the distribution is not normal, but rather Poisson or negative binomial then that distribution will be used.<br>This will be decided by selecting the best fitting model (i.e. that with the highest log-likelihood will be used)

Outcome	Residents' episodes of hospitalisation
Effect size	Mean difference in total number of hospitalisations per care home
Primary Analysis model	Linear regression model of the summary statistics for each home, the intervention group will be a fixed effect along with the number of staff.

Sensitivity analysis	None
Missing data	Care homes with missing data will be excluded from the analysis.
Other comments	If the distribution is not normal, but rather Poisson or negative binomial then that distribution will be used.<br>This will be decided by selecting the best fitting model (i.e. that with the highest log-likelihood will be used)

Outcome	Residents' mortality
Effect size	rate ratio
Primary Analysis model	Poisson regression of the number of deaths in each home the intervention group will be a fixed effect along with the number of residents at baseline
Sensitivity analysis	None
Missing data	Care homes with missing data will be excluded from the analysis.
Other comments	

6.3 Missing Data

Due to lack of individual participant data it is felt that no appropriate imputation model could be devised and hence no imputations are planned. However, we will look at the sensitivity of the results to missing data using the approach of White et al 2018, which uses pattern mixture models to allow for data to be not missing at random.

6.4 Additional analyses

Exploratory analyses

a) Vaccination clinics

Given the importance of the flu vaccination clinics the analysis of the primary and secondary outcomes will be repeated excluding any Care homes that did not have any vaccination clinics.

b) Other intervention components (intervention group only)

The other intervention components will be assessed at a care home level by splitting them into quartiles and presenting a boxplot of the vaccination rates in each category. The following components will be assessed:

i) the number of times a video is viewed by a care home divided by the number of staff at the care home;
ii) the number of posters in a care home

A test for trend using a Pearson correlation coefficient between the measure and the vaccination rate will be reported.

c) Excluding staff members who leave care home employment

If more than 10% of staff members leave the care home within 30 days of starting the study, then the analysis of the primary outcome will be repeated excluding these individuals.

Subgroup analyses

The ethnicity subgroup will be investigated by including an interaction term in the primary model; the individual estimates for each ethnicity group will be presented when the sample size allows (i.e. if >5% of participants are in the ethnicity group). An analysis reducing ethnicity to 'white' and 'non-white' will also be presented.

6.5 Safety analyses
None

6.5 Software
Stata v17.0 or later

7.0 References

White IR, Carpenter J, Horton NJ. A mean score method for sensitivity analysis to departures from the missing at random assumption in randomised trials. Statistica Sinica 2018;28:1985–2003.
