# Supplementary material for: Effectiveness of a theory-informed intervention to increase care home staff influenza vaccination rates: a cluster randomised controlled trial
Source: J Public Health (Oxf). 2025 Mar 30;47(2):246–57. doi: 10.1093/pubmed/fdaf023 (PMC12123319; doi:10.1093/pubmed/fdaf023)
Supplement: FluCare_TIDieR_checklist_fdaf023 [file flucare_tidier_checklist_fdaf023.docx]

**Supplementary file 1**

**Template for intervention description and replication (TIDieR) checklist**

| Item No | Item |
| --- | --- |
| Brief name | |
| 1 | Flucare intervention: A behaviour change intervention to facilitate care home staff influenza vaccination |
| Why | |
| 2 | The Flucare intervention is underpinned by behaviour change theory (the Theoretical Domains Framework (TDF)) and its linkage to the behaviour change techniques taxonomy version 1).  The five barriers (TDF domains) of care home staff influenza vaccination that the intervention was designed to address are:   1. Lack of staff time to obtain the influenza vaccine off site (environmental context and resources) 2. Lack of funding for the care home staff to obtain the influenza vaccine (environmental context and resources) 3. Care home staff perceived lack of need for the influenza vaccine (beliefs about consequences) 4. Peers do not obtain influenza vaccine (social influence) 5. Perception that the influenza vaccine is ineffective or causes disease (beliefs about consequences) |
| What | |
| 3 | Materials:  Six behaviour change techniques to address the five barriers were selected and operationalised into three intervention components in a previous study by care home staff:  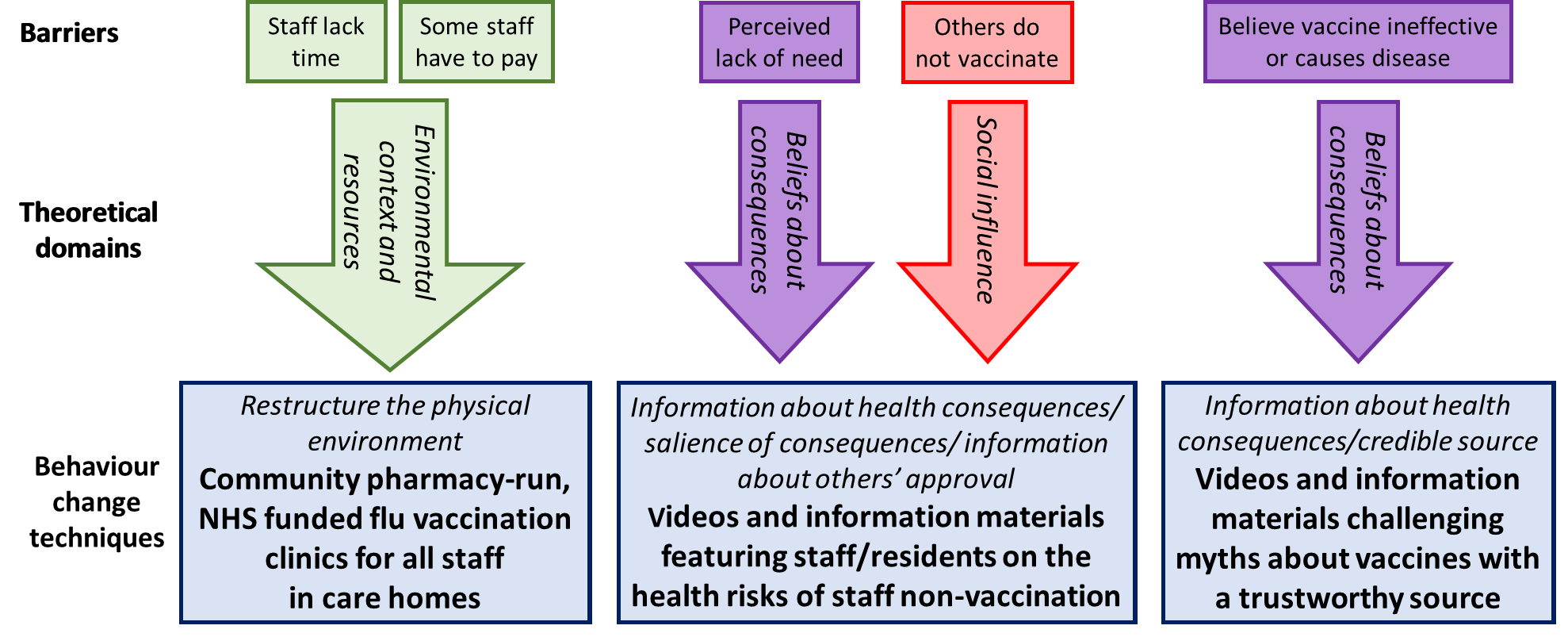  (Videos and posters not available due to risk of contamination in ongoing implementation) |
| 4 | Procedures:  Video lasting 3 minutes 55 seconds was of stakeholders (i.e. not actors) and was recorded in their place of work/residence e.g. care home, general practice. Videos and information materials were produced by a professional marketing company.  Following allocation to intervention, care home managers were emailed the link to the videos of stakeholders to share with staff during team meetings, and posted posters and leaflets (information materials A2 A3 A4 A5 sizes were used) promoting flu vaccination for distribution around the home. Community pharmacy staff paired with the care home were requested by the research team to liaise with the care home manager, agreeing suitable dates and times to deliver the flu vaccination clinics to be delivered in the care home at times convenient for staff. Community pharmacies were approached to provide clinics using a variety of methods e.g. contacting large multiples directly and networks of independent pharmacies. The clinics could be conducted alongside or separate to resident flu and/or covid vaccination sessions. |
| Who provided | |
| 5 | Community pharmacists providing the vaccination clinics were trained to administer the vaccines using their usual procedures. |
| How | |
| 6 | Videos were provided using an online link and care home managers were free to disseminate the link to their staff using their usual methods e.g. team meetings. Information materials were posted to the care home. Vaccination clinics occurred at the care home. |
| Where | |
| 7 | Videos were shown to staff at the care home and information materials were displayed in prominent areas, at the discretion of the care home manager, e.g. staff room. Vaccination clinics were provided at the care home at mutually convenient times for the pharmacy and care home teams. |
| When and how much | |
| 8 | Vaccination providers were asked to liaise with care home to arrange up to 3 clinics for staff to be given the opportunity to be vaccinated. Care home managers were asked to show the videos during staff meetings/Shift handovers and by sending out using their preferred communication method with staff, email/WhatsApp etc, They were given 7 days of the trial commencing in their care home and placed information materials in prominent locations like on the back of toilet doors. |
| Tailoring | |
| 9 | If the intervention was planned to be personalised, titrated or adapted, then describe what, why, when, and how  There was no tailoring to the content of the intervention.  Showing of videos and placing of information materials was at the care home manager’s discretion and the number and timings of vaccination clinics was a Joint agreement between the care home and the Vaccination provider. |
| Modifications | |
| 10 | No modifications were permitted. |
| How well | |
| 11 | A separate manuscript describing the methods for the process evaluation will be published. |
| 12 | A separate manuscript describing the methods for the process evaluation will be published. |
