# Supplementary material for: Effectiveness of a theory-informed intervention to increase care home staff influenza vaccination rates: a cluster randomised controlled trial
Source: J Public Health (Oxf). 2025 Mar 30;47(2):246–57. doi: 10.1093/pubmed/fdaf023 (PMC12123319; doi:10.1093/pubmed/fdaf023)
Supplement: Protocol_FluCare_Phase_3_v1_4_27Feb2023_fully_signed_fdaf023 [file protocol_flucare_phase_3_v1_4_27feb2023_fully_signed_fdaf023.pdf]

FluCare

FluCare Phase 3: Estimating the effectiveness and cost-effectiveness of a complex intervention to increase care home staff influenza vaccination rates.

Version	Version 1.4
Date	27 Feb 2023
Sponsor	University of East Anglia
Trial registration	ISRCTN 22729870
IRAS #	316820

Authorisation: Co-Chief Investigator

Name	Dr Amrish Patel
Role	Chief Investigator
Signature	
Date	10/3/23

Authorisation: Sponsor

Name	Professor David Wright
Role	Chief Investigator
Signature	
Date	13/3/23

Authorisation: Sponsor

Name	~~Phil Stasney~~ Tracy Moulton
Role	Sponsor Representative
Signature	
Date	31.3.23

Authorisation: CTU Director or Representative

Name	Matt Hammond
Role	CTU Deputy Director
Signature	
Date	29.03.23

Authorisation: Trial Statistician

Name	Allan Clark
Role	Trial Statistician
Signature	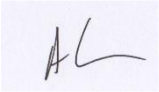
Date	28/03/2023

Authorisation: Trial Health Economist

Name	Adam P Wagner
Role	Trial Health Economist
Signature	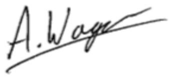
Date	28.03.2023

Table of Contents

1 Administrative information ... 1

1.1 Compliance ... 1

1.2 Sponsor ... 1

1.3 Structured trial summary ... 2

1.4 Roles and responsibilities ... 5

1.4.1 Protocol contributors ... 5

1.4.2 Role of trial sponsor and funders ... 6

1.4.3 Programme Management Group ... 6

1.4.5 Programme Steering Committee ... 7

1.4.6 Data Management Committee ... 8

1.4.7 Expert Advisory Panel ... 8

1.4.8 PPI Advisory Group ... 9

2 Trial diagram ... 10

3 Abbreviations ... 11

4 Glossary ... 11

5 Introduction ... 12

5.1 Background and rationale ... 12

5.1.1 Explanation for choice of comparators ... 12

5.2 Objectives ... 16

5.2.1 Study Within a Trial research questions (see Appendix 2) ... 16

5.3 Trial Design ... 17

6.1 Recruiting Site Selection ... 17

6.1.1 Study Setting ... 17

6.1.2 Recruiting Site/Investigator Eligibility Criteria ... 17

6.1.2 Participating Site approval and activation ... 17

6.3 Participants ... 17

6.3.1 Care Home Eligibility Criteria ... 17

6.3.1.1 Care Home selection ... 17

6.3.1.2 Care Home Inclusion Criteria ... 18

6.3.1.3 Care Home Exclusion Criteria ... 18

6.3.1.4 Care Home Co-enrolment Guidance ... 18

6.3.1.5 Screening Procedures and Pre-randomisation Investigations ... 18

6.3.2 Care Home Staff Eligibility Criteria ... 18

6.3.3 Care Home Resident Eligibility Criteria 18
6.3.4 Pharmacy / GP practice vaccination provider eligibility criteria (Intervention care homes only) 18
6.3.4.1 Pharmacy / GP practice selection criteria 18
6.3.4.2 Pharmacy / GP practice vaccination provider(s) inclusion criteria 19
6.3.1.3 Pharmacy / GP practice vaccination provider(s) Exclusion Criteria 19
6.4 Interventions 19
6.4.1 Arm A Usual Care 19
6.4.2 Arm B – Flu Vaccination Behaviour Change Intervention 19
6.4.5 Concomitant Care 20
6.4.7 Protocol Treatment Discontinuation 20
6.5 Outcomes 20
6.5.1 Primary Outcomes 20
6.5.2 Secondary Outcomes 20
6.5.3 Process Evaluation outcomes 21
6.5.4 Health economic outcomes 21
6.5.5 Study Within A Trial (SWAT) outcomes 21
6.5.5 Process Evaluation 21
6.5.5.1 Process evaluation objectives: 21
6.5.5.2 Process evaluation data collection methods 21
6.6 Participant Timeline 24
6.6.1 Care Home Manager and Staff Assessments 24
6.6.3 Early Stopping of Follow-up 25
6.6.4 Participant Transfers 25
6.6.5 Loss to Follow-up 26
6.6.6 Trial Closure 26
6.7 Sample Size 26
6.8 Recruitment, Retention and Data Completeness 27
6.8.1 Recruitment 27
6.8.2 Retention 27
6.8.3 Data Completeness 27
6.9 Assignment of Intervention 28
6.9.1 Allocation 28
6.9.1.1 Sequence generation 28
6.9.1.2 Allocation concealment mechanism 28

6.9.1.3 Allocation Implementation 28
6.9.2 Blinding 28
6.10 Data Collection, Management and Analysis 28
6.10.1 Data Collection Methods 28
6.10.2 Data Management 29
6.10.3 Non-Adherence and Non-Retention 30
6.10.4 Statistical Methods 30
6.10.4.1 SWAT Statistical Methods 30
6.10.5 Health Economic Methods 31
6.10.6 Process Evaluation Methods 32
6.10.6.1 Analysis of implementation outcomes 32
6.10.6.2 Analysis of MAQ data 32
6.10.6.3 Analysis of interviews 32
6.11 Data Monitoring 33
6.11.1 Data Monitoring Committee (DMC) 33
6.11.2 Interim Analyses 33
6.11.4 Quality Assurance and Control 33
6.11.4.1 Risk Assessment 33
6.11.4.2 Central Monitoring at NCTU 34
6.11.4.3 On-site Monitoring 34
6.11.4.4 Trial Oversight 34
6.11.4.4.2 Programme Management Group 34
6.11.4.4.3 Independent Programme Steering Committee 34
6.11.4.4.4 Data Monitoring Committee 35
6.11.4.4.5 Trial Sponsor 35
7 Ethics and Dissemination 35
7.1 Research Ethics and Health Research Authority Approval 35
7.2 Competent Authority Approvals 35
7.3 Other Approvals 35
7.4 Amendments 36
7.5 Consent 36
7.6 Confidentiality 37
7.7 Declaration of Interests 38
7.8 Indemnity 38
7.9 Finance 38

7.10 Archiving 38
7.11 Access to Data 38
7.12 Ancillary and Post-trial Care 38
7.13 Publication Policy 39
7.13.1 Trial Results 39
8 Protocol Amendments 40
9 References 41
10 Appendix 1 – Logic model 1
11 Appendix 2 – Study Within A Trial (SWAT) 1

Table of Figures

Figure 1 Relationship between behaviour change techniques, barriers and theory 14

1 Administrative information

This document was constructed using the Norwich Clinical Trials Unit (NCTU) Protocol template Version 4.1. It describes the FluCare trial, sponsored by University of East Anglia and co-ordinated by NCTU.

It provides information about procedures for entering participants into the trial, and provides sufficient detail to enable: an understanding of the background, rationale, objectives, trial population, intervention, methods, statistical analyses, ethical considerations, dissemination plans and administration of the trial; replication of key aspects of trial methods and conduct; and appraisal of the trial's scientific and ethical rigour from the time of ethics approval through to dissemination of the results. The protocol should not be used as an aide-memoire or guide for the treatment of other patients. Every care has been taken in drafting this protocol, but corrections or amendments may be necessary. These will be circulated to registered investigators in the trial. Sites entering participants for the first time should confirm they have the correct version through a member of the trial team at NCTU.

NCTU supports the commitment that its trials adhere to the SPIRIT guidelines. As such, the protocol template is based on the Standard Protocol Items: Recommendations for Interventional Trials (SPIRIT) 2012 Statement for protocols of clinical trials [1]. The SPIRIT Statement Explanation and Elaboration document [2] can be referred to, or a member of NCTU Protocol Review Committee can be contacted for further detail about specific items.

1.1 Compliance

The trial will be conducted in compliance with the approved protocol, the Declaration of Helsinki (2008), the principles of Good Clinical Practice (GCP) as laid down by the Commission Directive 2005/28/EC with implementation in national legislation in the UK by Statutory Instrument 2004/1031 and subsequent amendments, the UK Data Protection Act, and the UK Policy Framework for Health and Social Care Research, and other national and local applicable regulations. Agreements that include detailed roles and responsibilities will be in place between participating sites and NCTU.

Participating sites will inform NCTU as soon as they are aware of a possible serious breach of compliance, so that NCTU can fulfil its requirement to report the breach, if necessary, within the timelines specified in the UK Clinical Trials Regulations (currently 7 days). For the purposes of this regulation a 'serious breach' is one that is likely to affect to a significant degree:

- The safety or physical or mental integrity of the subjects in the trial, or
- The scientific value of the trial.

1.2 Sponsor

University of East Anglia is the trial sponsor and has delegated responsibility for the overall management of the FluCare trial to the Co-Chief Investigators and NCTU. Queries relating to sponsorship of this trial should be addressed to Dr Amrish Patel or via the trial team. University of East Anglia is data controller.

1.3 Structured trial summary

Primary Registry and Trial Identifying Number	ISRCTN to be confirmed
Date of Registration in Primary Registry	to be confirmed
Secondary Identifying Numbers	RIN R209939<br><br>IRAS number: 316820
Source of Monetary or Material Support	National Institute of Health Research Public Health Research Funding Stream
Sponsor	University of East Anglia
Contact for Public Queries	Flu.care@uea.ac.uk
Contact for Scientific Queries	Dr Amrish Patel<br>Associate Professor in Economics<br>(School of Economics)<br>University of East Anglia,<br>Norwich Research Park,<br>Norwich,<br>NR4 7TJ<br><br>Email: Amrish.Patel@uea.ac.uk<br>Telephone: Tel: 01603 597644
Short Title or Acronym	FluCare Study
Scientific Title	FluCare Study: Estimating the effectiveness and cost-effectiveness of a complex intervention to increase care home staff influenza vaccination rates.
Countries of Recruitment	England
Health Condition(s) or Problem(s) Studied	The World Health Organization (WHO) recommends that at least 75% of health and social care staff are vaccinated for flu. Whilst the target has been met for healthcare staff in England, the figure was last reported at only 25% for social care staff.
Intervention(s)	**Usual Care**

	Arm A: Usual care with baseline, monthly and end of study data collection<br><br>**Intervention:**<br><br>Arm B: A multi-component intervention, addressing the barriers to care home staff flu vaccine uptake, comprising online videos and supporting information materials (including posters and leaflets) and incentives. Baseline, monthly and end of study data collection
Key Inclusion and Exclusion Criteria	**Care Homes**<br><br>Inclusion criteria:<br><br>• Long stay for older residents or dementia registration<br>• self-reported staff vaccination rate <40%<br>• signed up to, or willing to sign up to the DHSC Capacity Tracker and willing to provide weekly updates on flu vaccine status of staff and residents<br><br>Exclusion criteria<br><br>• Fewer than 10 staff members<br>• Participated in Flucare feasibility study<br><br>**Care Home Staff**<br><br>• All staff working at the care home for questionnaire completion only<br><br>**Care Home Residents**<br><br>• All residents irrespective of whether they are permanent or respite residents for aggregate data
Study Type	Two-arm, parallel, randomised controlled trial of the effectiveness and cost-effectiveness of a multi-component care home staff focused influenza vaccination engagement intervention compared to usual care, with embedded process evaluation.<br>Homes will be randomised with stratification by the percentage of staff identifying as non-white following consent.

Date of First Enrolment	August 2022
Target Sample Size	78 Care Homes
Outcome(s)	Primary Outcome:<br>Total number of staff vaccinated in a flu season over total number of staff employed at any point throughout that flu season (all directly contracted staff (care staff, cleaners, cooks, administrative staff))<br><br>Secondary Outcomes:<br>staff flu vaccination rate at end of November;<br>number of staff sick days;GP and nurse visits to care home;<br>care home resident hospitalisations;<br>care home resident mortality.<br><br>Health Economic Outcomes:<br>Intervention delivery costs<br><br>Process Evaluation Outcomes:<br>summarise the amount (or dose) of the intervention delivered to each home;<br>examine and define the mechanisms of action, adaptions and variations across care homes.

1.4 Roles and responsibilities

These membership lists are correct at the time of writing; please see terms of reference documentation in the TMF for current lists.

1.4.1 Protocol contributors

Name	Affiliation	Role [individuals who contribute substantively to protocol development and drafting should have their contributions reported]
Dr Amrish Patel	UEA	Co-Chief Investigator
Professor David Wright	University of Leicester	Co-Chief Investigator
Dr Erika Sims	UEA	Clinical Trial Operations
Dr Alys Griffiths	University of Liverpool	PPI academic lead
Professor Richard Holland	University of Leicester	Consultant in public health
Dr Linda Birt	University of Leicester	Process Evaluation and Qualitative Analysis Lead
Dr Sion Scott	University of Leicester	Behavioural science and qualitative analysis
Dr Adam P Wagner	UEA	Health economics lead
Professor Andy Jones	Norfolk County Council	Design and implementation of intervention evaluation
Dr Allan Clark	UEA	Statistician
Mr Tony Dean	Norfolk Local Pharmaceutical Committee	Advise on configuring and commissioning pharmacy services and implementation
Mr Luke Cook	Askham Community Village	PPI and advise on care home context
Dr Liz Jones (LJ-PPI)	PPI	PPI representative (relative of care home resident); Expert Panel Lead
Jeanette Blacklock	University of Leicester	Care home research delivery

Dr Thando Katangwe-Chigamba	UEA	Process Evaluation
Po Ruby	UEA	Care home research delivery & Process Evaluation
Mrs Veronica Bion	UEA	NCTU Trial Manager
Mrs Jennifer Pitcher	UEA	Care home research delivery

1.4.2 Role of trial sponsor and funders

Name	Affiliation	Role
Phil Stastney	UEA	Sponsor Representative
Clare Symms	Norfolk and Waveney CCG	Host Representative

1.4.3 Programme Management Group

Name	Affiliation	Role and responsibilities
Dr Amrish Patel	UEA	Co-Chief Investigator
Professor David Wright	University of Leicester	Co-Chief Investigator
Mr Matthew Hammond	UEA	Deputy Director of the Norwich Clinical Trials Unit
Dr Erika Sims	UEA	Clinical Trial Operations
Dr Alys Griffiths	University of Liverpool	PPI academic lead
Professor Richard Holland	University of Leicester	Consultant in public health
Dr Linda Birt	University of Leicester	Process Evaluation and Qualitative Analysis Lead
Dr Sion Scott	University of Leicester	Behavioural science and qualitative analysis
Dr Adam Wagner	UEA	Health economics lead
Professor Andy Jones	Norfolk County Council	Design and implementation of intervention evaluation

Dr Allan Clark	UEA	Statistician
Mr Dele Famokunwa	DHSC	Flu vaccination in care homes specialist – advisor for government policy
Mr Tony Dean	Norfolk Local Pharmaceutical Committee	Advice on configuring and commissioning pharmacy services and implementation
Mr Luke Cook	Askham Community Village	PPI and advice on care home context
Dr Liz Jones (LJ-PPI)	PPI	PPI representative (relative of care home resident) and PPI Lead
Jeanette Blacklock	University of Leicester	Senior Research Associate
Dr Thando Katangwe-Chigamba	UEA	Senior Research Associate (Process Evaluation)
Carys Seeley	UEA	Research Associate
Jennifer Pitcher	UEA	Senior Research Associate
Mr Faisal Alsaif	UEA	Post-graduate PhD Student
Ms Cecile Guillard	UEA	NCTU Data Programmer
Mr Martin Pond	UEA	NCTU Data Manager
Mrs Veronica Bion	UEA	NCTU Trial Manager
Amber Hammond	UEA	NCTU Trial Assistant
Gosia Majsak-Newman	Norfolk & Waveney CCG	R & D Officer

1.4.5 Programme Steering Committee

Name	Affiliation	Role and responsibilities
Professor Michael Dewey	Kings College London	Independent Chair and Independent Statistician
Dr Tim Davis	NHS England and NHS Improvement	Independent Public Health Specialist

Professor Stephen Byrne	University College Cork, Ireland	Independent Trialist
Dr Amrish Patel	UEA	Co-Chief Investigator
Professor David Wright	Leicester	Co-Chief Investigator
Professor Martin Green	Care England	Independent Stakeholder Representative; Chief Executive Officer;
Professor Julienne Meyer	National Care Forum	Independent Stakeholder Representative, Research and Development Advisor;
Clare Symms	Host Organisation	Head of Research Management, Finance and PPI, Observer
Phil Stastney	UEA	Sponsor Representative, Observer
Dr Allan Clark	UEA	Statistician
Dr Erika Sims	UEA	NCTU Research Lead – Complex Interventions
Dr Krystal Warmoth	University of Hertfordshire	Independent; Behavioural Scientist
Ms Helen Jackson	PPI	Independent PPI member
Ms June Sanson	PPI	Independent PPI member

1.4.6 Data Management Committee

Name	Affiliation	Role and responsibilities
Ms Nicky Perry	Brighton & Sussex CTU, University of Sussex	Trialist, Independent Chair
Professor Julius Sim	University of Keele	Independent Statistician
Vacancy		

1.4.7 Expert Advisory Panel

Name	Affiliation	Role and responsibilities
Liz Jones (LJ-EAP)	National Care Forum (NCF)	Policy Director at NCF
David James	CQC	Head of Adult Social Care Policy
Tracey Thornley	Boots UK	Manager of Contract Framework and Outcomes
Emma Smith	Wakefield Council	Health Protection Manager
Chris Pearson	HC-One	Flu Campaign Manager
Care Home Staff (tba)		
Care Home Staff (tba)		

1.4.8 PPI Advisory Group

Name	Affiliation	Role and responsibilities
Dr Liz Jones (LJ-PPI)	PPI Lead	PPI representative (relative of care home resident); PPI co-Lead
Dr Alys Griffiths	University of Liverpool	PPI academic lead
Alison Bryant	PPI member	PPI representative
Robert T Bryant	PPI member	PPI representative
Hilary Tetlow	PPI member	PPI representative
Hilary Garrett	PPI member	PPI representative
Keith Holt	PPI member	PPI representative
Saima Gul	PPI member	PPI representative
Saiqa Ahmed	PPI member	PPI representative

2 Trial diagram

*Twenty homes, 10 intervention and 10 usual care will receive the Mechanism of Action Questionnaire (MAQ). These twenty plus an additional two intervention homes will participate in the process evaluation. #UDM – Understanding Decision Making questionnaire is part of the Study Within a Trial and will be given to homes not receiving the MAQ.

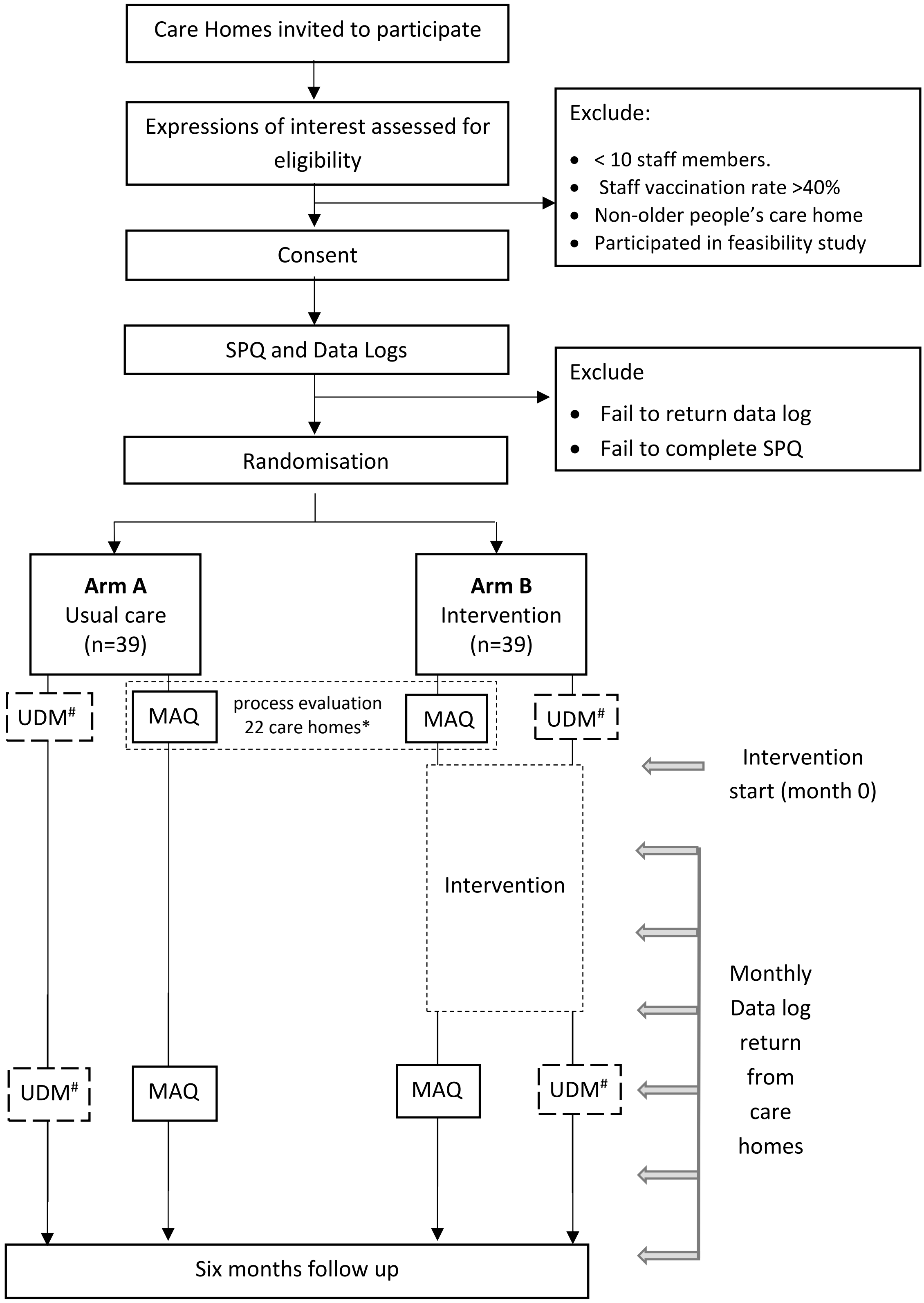

3 Abbreviations

AE	Adverse Event
BCT	Behaviour Change Techniques
CH	Care Home
CI	Chief Investigator
CRF	Case Report Form
DMC	Data Management Committee
GCP	Good Clinical Practice
HRA	Health Research Authority
ITT	Intention to Treat
MAQ	Mechanism of Action Questionnaire
NCTU	Norwich Clinical Trials Unit
PI	Principal Investigator
PID	Participant Identification Number
PIS	Participant Information Sheet
PMG	Programme Management Group
PSC	Programme Steering Committee
QA	Quality Assurance
QC	Quality Control
QMMP	Quality Management and Monitoring Plan
R&D	Research and Development
REC	Research Ethics Committee
SAE	Serious Adverse Event
SAP	Statistical Analysis Plan
SSA	Site Specific Approval
SWAT	Study within a Trial
TDF	Theoretical Domains Framework
TMF	Trial Master File
TMT	Trial Management Team
ToR	Terms of Reference
UEA	University of East Anglia
UDM	Understanding Decision Making questionnaire

4 Glossary

Social Care Workers – for the purpose of this project, social care workers are care home staff.

5 Introduction

5.1 Background and rationale

Each year seasonal influenza (flu) causes 17,000 UK deaths [3]. This creates a major risk for older residents of care and nursing homes [4], [5]. Vaccinating care staff is known to mitigate against this [4], [6]–[9].

Evidence suggests a linear relationship between staff flu vaccine uptake and resident health outcomes [10], [11]. Higher staff flu vaccination rates reduce residents' flu-like-illness, hospitalisation and mortality [4], [6]–[9]. Staff health improves [12], implying fewer sick days [13], improved care continuity and quality [14], lower staff cover costs [15], and more financially viable homes. The World Health Organization (WHO) recommends that at least 75% of health and social care staff are vaccinated for flu [16]. Whilst the target has been met for healthcare staff in England [17], the figure was last reported at only 25% for social care staff [18]. Our survey (415 care home staff respondents), found a 38% vaccination rate for the 2019-20 flu season [19]. For 2020-21, a 34% flu vaccination rate was reported for care home staff (NHS Capacity Tracker [20]), despite the COVID pandemic.

Policy initiatives based on the existing (mostly healthcare sector) evidence have been enacted (e.g. NHS funded vaccines; pharmacist-led vaccinations; evidence-based flu campaign guidelines [21]–[23]) with little effect on care home staff uptake. Despite a 2020 policy change allowing pharmacists to administer NHS flu vaccines to staff in care homes, few do so due to the costs involved. Several policy initiatives have attempted to increase flu vaccine uptake in care home staff with limited effect (e.g. NHS funded vaccines; pharmacy vaccinations; flu campaign guidelines [21]–[23]). These initiatives usually address one barrier to vaccination at a time and do not approach the problem in a holistic manner. An intervention designed to overcome all barriers and use all enablers simultaneously to maximise effectiveness is required.

The UK's COVID vaccination programme has been very successful in part due to the high perceived need for vaccination. Over time COVID risks will likely become normalised and the perceived urgency of booster vaccinations is likely to be significantly lower. Furthermore, COVID lockdowns and social distancing mean that a severe resurgence of flu is likely as immunity is lower than usual and pressures on the virus mean a more transmissible strain emerging is more probable [24]. The outcomes of this research project will be used to inform the design and delivery of future COVID booster vaccination programmes, especially if the flu and COVID vaccinations are combined [25].

We have developed an intervention to support flu vaccination uptake for care home staff, in line with MRC guidance [26], and underpinned by behavioural science using the Theoretical Domains Framework (TDF)[27], a systematic review and narrative synthesis of the literature (Prospero: CRD42021248384) plus extensive stakeholder engagement. We propose to evaluate this intervention against usual care.

5.1.1 Explanation for choice of comparators

The 2018 NICE evidence review on increasing flu vaccination uptake [21] identified a number of areas lacking evidence: (i) The effectiveness and cost-effectiveness of interventions to increase uptake for carers (including care home staff); (ii) The effectiveness and cost-effectiveness of community-based flu vaccination provision models (e.g. pharmacy) and (iii) How information should be tailored/delivered to increase vaccine uptake.

Systematic reviews and meta-analyses on the effectiveness of interventions aiming to increase health/social care worker flu vaccine uptake [21], [28]–[30] suggest that most existing studies examine healthcare workers (e.g. NICE review, only 5 of 31 studies were on care homes and none were UK-based [21]).

In 2017-18, Wakefield Council commissioned two pharmacies to proactively contact 27 homes and offer in-home staff vaccination clinics [31]; vaccination rates rose from 10% to 40%. Our research will: determine whether a more optimised intervention (e.g., regular clinics accounting for shift-work and financial incentives for homes) can achieve the WHO's 75% target; provide evidence that is more detailed (by including a process evaluation), and robust (via a RCT design); determine how delivery costs fall across different stakeholders, and whether resulting benefits lead to cost-savings. NICE evidence review found no cost- effectiveness studies on interventions that increase staff access to flu vaccination [21].

While there is limited evidence that financial incentives for staff can increase vaccine uptake [32], we have not identified any studies estimating the effectiveness of an intervention containing financial incentives for homes to encourage vaccination.

By combining a range of interventions into our multi-component intervention we provide evidence for a new more holistic intervention specifically designed for UK care home staff. There are no trials registered on the WHO International Clinical Trials Registry Platform [33] exhibiting significant overlap with our proposed research.

From collation of the evidence obtained from our narrative synthesis, survey and qualitative work, we identified five main individual-level barriers to flu vaccination (2 non-cognitive and 3 cognitive):

1. Access (non-cognitive): Staff lack time to access vaccine through traditional routes. Although GPs and pharmacists are permitted to vaccinate staff in care homes, most do not do so (e.g., Boots UK, >50% care home market) as it is not cost-effective given the current commissioning model. Care home staff working shifts and nights are thus expected to make their own way to GP practices and pharmacies for vaccination. This is a major barrier. Care home staff often cite this barrier and suggest the natural solution: "The single most helpful action would be to offer flu vaccination in-house" [19] [. "Convenience" is one of the three barrier categories that comprise the WHO's 3Cs model of vaccine hesitancy [34].

2. Cost (non-cognitive): Some care home staff (e.g. agency) are required to pay for vaccine. Staff directly employed by a home and closely involved in resident care are entitled to an NHS flu vaccination [23]. Other staff (e.g. agency/temp staff, 10% of the workforce [35]) are not entitled to a free NHS vaccination. Cost is a well-known vaccine uptake barrier [34], [36].

3. Perceived lack of need (cognitive): Staff perceive no need for the vaccine as they are healthy. A large share of non-vaccinating staff cite this as the reason for non-vaccination (e.g. 23%- 67% [19] [37] [38]). "Complacency" is another barrier category in the WHO's 3Cs model[34].

4. Vaccine beliefs (cognitive): Beliefs that vaccine is either ineffective or causes disease. A large proportion of non-vaccinating staff cite these reasons for non-vaccination (e.g. 34-60%[19] [39] [38] [40]). "Confidence" (e.g. in vaccine effectiveness) is again another barrier category of WHO's 3Cs model of vaccine hesitancy [34].

5. Peer influence (cognitive): Negative influence of non- or anti-vaccination movement. Non-vaccinated staff often remark how very few other staff get vaccinated in their workplace[19]. Peer effects and norms are important determinants of vaccine uptake[37].

Figure 1 provides our mapping of the five barriers to care home staff vaccination to the TDF domains which require addressing. Using the mapping table by Cane et al. [41], we identified 31 potentially appropriate Behaviour Change Techniques (BCTs), the active ingredients of behaviour change interventions, with evidence for addressing the TDF domains in Figure 1. We subsequently convened a Nominal Group Technique stakeholder consensus study [42] with 13 care home staff and managers to develop an intervention. Stakeholders selected from the list of BCTs, those which met the APEASE criteria (affordable, practicality, effectiveness, acceptability, side-effects, equity) for addressing the barriers [43].

Figure 1 Relationship between behaviour change techniques, barriers and theory

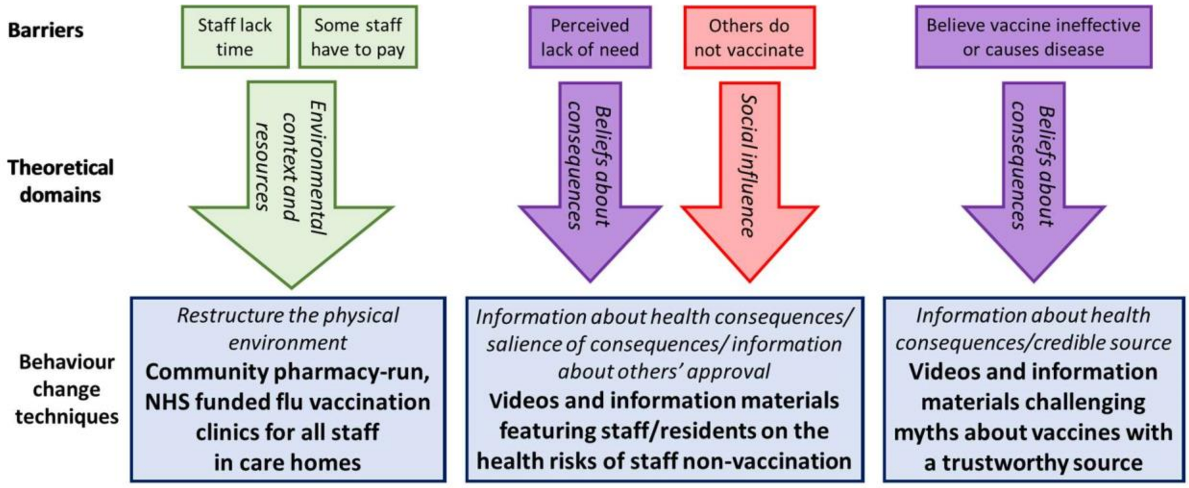

After selecting BCTs to include in the intervention, Nominal Group Technique stakeholders proceeded to characterise how each BCT may be operationalised in practice. This characterisation was refined by public and patient involvement (care home residents and relatives) and stakeholder input to arrive at the following:

BCT: Restructure the physical environment: A pharmacy will offer NHS funded flu vaccination clinics to all staff (inc. agency) in homes. Stakeholders identified that clinics should be run by the pharmacy currently supplying the home's resident medication to leverage the existing trusted relationship. PPI input suggested that several clinics would have to be run at convenient times to account for shift-/night-work and maximise access.

BCTs: Information about health consequences, salience of consequences and information about others' approval (operationalised together): Information on the health risks of low staff vaccine uptake featuring staff and residents. Stakeholders believed that an engaging 5-10 minute video would work best, with residents and vulnerable staff (older and younger) discussing serious health risks to them arising from poor staff vaccine uptake and how vaccination protects everyone. They also believed the videos should be integrated into existing staff processes (e.g., handovers, inductions or staff apps) to ensure engagement and that posters or other information materials could reinforce the main images/messages. PPI highlighted that materials should reflect staff cultural diversity (i.e. multi-

lingual with a range of socio-demographics), particularly given low vaccine uptake in BAME communities [44].

BCTs: Information about health consequences and credible source (operationalised together): Information from a trustworthy source e.g., General Practitioner, challenging the myths about vaccines. Stakeholders identified a similar format (i.e., short video supported by information materials) and developed some of the myths to be challenged. These included: that the vaccine is dangerous to pregnant women and that it causes flu.

While our intervention targets staff level behaviour change, it is widely recognised that for staff to undertake a behaviour, they must feel it aligns with the priorities of their organisation [45]. Employer encouragement is a known enabler for staff vaccination [21], [46], [47]. Care homes receive staff flu campaign guidance (NHS [23]; PHE [22]) based on a NICE evidence review [21] and are required to facilitate staff vaccination. Implementation is variable: 16% of our care home staff survey respondents said their employer did not promote vaccination; a further 10% made statements like: “I noticed a poster but there’s no encouragement” [19].

Our intervention (Figure 1) is thus complemented by two organisational-level strategies:

1. Regular vaccine uptake monitoring of care homes
2. Financial incentives for care homes with staff vaccination rate >70%

Evidence suggests that incentivisation, monitoring and feedback facilitate organisational-level support for behaviour change (e.g. CQUIN financial incentives in the NHS increasing healthcare staff flu vaccine uptake [48]). The use of incentive payments was viewed as particularly powerful by sector leaders as it signalled equity between health (NHS) and social care. Many local authorities pay premia to homes to incentivise care quality in general[49].

Finally, several care home managers reported shortages of vaccine supplies [50]. In-home clinics should mean staff get vaccinated earlier (i.e., before shortages occur) and our intervention pharmacies and/or GP Practices will be required to withhold sufficient vaccines to support vaccination of any new members of care home staff starting during the intervention period.

The FluCare feasibility trial undertaken during the 2021/2022 flu season, confirmed that care homes and vaccination providers (GPs and Pharmacists) could be successfully recruited and were willing to participate. The feasibility study informed the frequency of data collection and design of the control arm. While the frequency of data collection (monthly versus end of study), did not influence the uptake of flu vaccination in the control arm, monthly data collection was preferred by sites. Although the provision of posters and leaflets appeared to have a small but limited effect. Strategies to improve data collection and data were also identified and used to inform the design of this definitive trial (phase 3) of the FluCare intervention versus usual care in care homes in England.

We will conduct a Study Within a Trial (SWAT) the aim of understanding care home staff’s decision-making characteristics via a short incentivised online survey. Economists often think of individuals’ choices as the outcome of rational decision-making processes. For example, weighing up the costs and benefits of an action to determine whether to undertake it. While some of these costs/benefit might be financial, they are often not. For instance, when deciding whether to get vaccinated, one can think of the “warm glow” you receive from knowing that you are protecting your family as a non-pecuniary benefit. Behavioural economists have developed a range of simple incentivised choice tasks that can be used to elicit and measure such decision-making characteristics. The data collected in this pilot

study will be used to conduct a mediation analysis, providing insights about the “type” of staff that the FluCare intervention does/does not work particularly well for.

5.2 Objectives

The overall objectives of this study are to:

1. Estimate the effect of the intervention on staff vaccination rates (primary outcome) and secondary outcomes identified in the logic model (e.g., residents’ morbidity and mortality; Appendix 1)
2. Explore the economic impact of the intervention (e.g., cost per vaccination percentage point increase)
3. Relate variations in intervention fidelity to intervention effectiveness (in an embedded process evaluation)

Specific process evaluation objectives are:

1. Generate suggestions for wider implementation of the intervention to other homes
2. To describe the intervention as delivered in terms of dose and fidelity, including adaptations and variations across care homes
3. To explore the effects of individual intervention components on the primary outcomes.
4. To investigate the mechanisms of impact.
5. To describe the perceived effectiveness of relevant intervention components (including videos, leaflets, posters and flu clinics) from participant (care home manager, care home staff and flu clinic providers) perspectives.
6. To describe the characteristics of care homes and participants to assess reach.

5.2.1 Study Within a Trial research questions (see Appendix 2)

The research questions:

A. Does the effectiveness of the behaviour change intervention vary by staff member’s risk-preferences?
B. Does the effectiveness of the behaviour change intervention vary with staff member’s prosociality?
C. Does the effectiveness of the behaviour change intervention vary by a staff member’s beliefs about other’s vaccination behaviour?

We will also address an additional research question which may shed light on whether the intervention works by affecting any of the decision-characteristics under study:

D. Does the behaviour change intervention change staff’s risk-aversion, prosociality or beliefs about other’s vaccination behaviour?

5.3 Trial Design

This is a low risk two arm, open label, definitive effectiveness and cost-effectiveness trial of FluCare, a behaviour change intervention designed to improve uptake of influenza vaccination by staff in care homes in England, compared to usual care, with an embedded process evaluation

The design of the trial was informed by a 5-arm feasibility trial conducted in 10 care homes. The feasibility trial confirmed that the usual care (Arm A) and intervention (Arm B) arms in this study will include baseline, monthly/end of study data collection and the intervention (Arm B) will also include videos, posters and leaflets.

6.1 Recruiting Site Selection

The trial sponsor has overall responsibility for site and investigator selection and has delegated this role to the CI and NCTU.

6.1.1 Study Setting

Community based private, charity, corporate or local authority care homes in England.

6.1.2 Recruiting Site/Investigator Eligibility Criteria

UEA will be the single recruiting site for this trial. Care Homes, GP practices and Pharmacies will be approached via Care Home networks, regulatory agencies and Care Home organisations to provide Expressions of Interest, but consent will be taken by the central research team electronically.

The Site Investigator for UEA is Dr Amrish Patel, CI for the FluCare project and grant holder.

6.1.2 Participating Site approval and activation

Participating sites are Care Homes, GP practices and Pharmacies, which will hereafter be referred to as sites.

Sites will be required to complete and sign an Organisation Information Document (OID) to confirm prior to providing consent to participate. Following confirmation of Sponsor approval, the site will be able to provide consent to participate. As the site is a participant in the study, recruitment to participate constitutes site activation.

6.3 Participants

6.3.1 Care Home Eligibility Criteria

6.3.1.1 Care Home selection

There will be NO EXCEPTIONS (waivers) to eligibility requirements at the time of allocation. Questions about eligibility criteria should be addressed PRIOR to attempting to randomise the participant. That said, it is known that multi-component interventions are needed to increase vaccine uptake [21], [34]. Offering vaccination opportunities in homes is associated with higher staff uptake [51], [52] Existing RCT evidence on interventions containing on site vaccination focus on specific types of home: nursing [9], [53]; a large private group [6] ; working with a particular pharmacy [54]; one geographic area [55]. The care home eligibility criteria have been carefully considered to maximise the generalisable evidence of intervention effectiveness by including all homes with low staff vaccination rates.

Care homes not meeting the criteria should not be entered into the trial to ensure that the trial results can be appropriately used to make future decisions for other behaviour change interventions. It is therefore vital that exceptions are not made to these eligibility criteria.

Care homes will be considered eligible for enrolment in this trial if they fulfil all the inclusion criteria and none of the exclusion criteria as defined below. Participating Care Homes will receive £500 for costs associated with facilitating the research including data collection.

6.3.1.2 Care Home Inclusion Criteria

- Registered to provide care for older residents, which may include people with dementia
- Self-reported staff vaccination rate <40%
- Must be signed up or willing to sign up to the DHSC Capacity Tracker and willing to submit weekly updates on flu vaccine status of staff and residents

6.3.1.3 Care Home Exclusion Criteria

- Fewer than 10 staff members (as insufficient qualitative and quantitative data likely to be produced).
- Participated in the FluCare feasibility trial

6.3.1.4 Care Home Co-enrolment Guidance

Care homes participating in existing trials of behaviour change interventions are ineligible to participate in FluCare. Care Home residents and staff may participate in trials of COVID-19 treatments.

6.3.1.5 Screening Procedures and Pre-randomisation Investigations

Written informed consent to enter and be randomised into the trial must be obtained from the Care Home Manager or Owner after confirmation of the eligibility criteria, explanation of the aims, methods, benefits and potential hazards of the trial and **BEFORE** any trial-specific procedures.

6.3.2 Care Home Staff Eligibility Criteria

- All staff (permanent, agency, voluntary) working at the care home at any time from randomisation to end of follow-up are eligible to participate

6.3.3 Care Home Resident Eligibility Criteria

- Residents (permanent or receiving respite care) living at the care home at any time from randomisation to end of follow-up will be included in the aggregate data

6.3.4 Pharmacy / GP practice vaccination provider eligibility criteria (Intervention care homes only)

6.3.4.1 Pharmacy / GP practice selection criteria

Care Homes consented to participate will be requested to provide information on their GP practice and/or pharmacy provider(s) to establish willingness to provide a vaccination service for the purpose of the trial, including out of hours provision. A preference to which provider to contact first will be informed by feedback from the care home with respect to local organisational structure and service provision. A flu vaccination service provided jointly by a GP practice and a pharmacy would be permitted.

6.3.4.2 Pharmacy / GP practice vaccination provider(s) inclusion criteria

- Willing to provide flu vaccinations within the care home to care home staff (permanent, agency, voluntary) and residents who were not vaccinated under the usual arrangements.
- Have adequate staff available to provide a flu vaccination service within the care home, including out of hours and for new starters.

6.3.1.3 Pharmacy / GP practice vaccination provider(s) Exclusion Criteria

- Unwilling to retain a small amount of vaccinations for the purposes of care home staff new starters appearing during the intervention period. Number required to be advised by the related home.

6.4 Interventions

6.4.1 Arm A Usual Care

Usual care will include baseline, monthly and end of study data collection. The Care Home manager/owner will be aware that the care home is participating in the trial, but no additional information will be provided to staff. Outcome data will be requested by the research team at baseline, on a monthly basis, and at the end of the study, to confirm data quality with feedback to the Care Home manager should issues be identified.

6.4.2 Arm B – Flu Vaccination Behaviour Change Intervention

The multi-component intervention will comprise of:

- Online videos of stakeholders (GP, Residents and care home staff) and supporting information materials (including posters and leaflets)
- Care home incentive scheme comprising of £850 incentive if more than 70% of care home staff receive a flu vaccination as reported on the Department of Health and Social Care Capacity Tracker and in Care Home Staff log
- GP and/or pharmacy vaccination provision comprising of up to 4 vaccination clinics organised around care home shifts
- Outcome data will be requested by the research team at baseline, on a monthly basis, and at the end of the study, to confirm data quality with feedback to the Care Home manager should issues be identified.

6.4.5 Concomitant Care

Care Home staff will be able to access NHS care via their usual GP and/or pharmacy provider. Should a member of staff in the intervention home prefer to receive their flu vaccination via their own GP or local pharmacy provider, this is permitted and will be captured in the care home staff log.

6.4.7 Protocol Treatment Discontinuation

<u>Care Home Managers / Owners</u>

In consenting to the trial, care home managers/owners are consenting to provide anonymised data on their staff engagement with flu vaccination and aggregate resident data. For intervention Care Homes, managers/owners will be responsible for distributing intervention materials to their staff. For a sample of intervention and control Care Homes, managers/owners will be asked to send invites to staff members to participate in questionnaire completion.

Should a care home go into CQC-defined special measures during the trial, this would not be a valid reason, in itself, for discontinuing participation.

As participation in the trial is entirely voluntary, the Care Home Manager/ Owner may choose to discontinue trial treatment at any time without penalty or loss of benefits to which they would otherwise be entitled. Although not obliged to give a reason for discontinuing their trial treatment, a reasonable effort should be made to establish this reason, whilst remaining fully respectful of the Care Home Manager / Owner's rights.

Care Home Manager / Owners who discontinue protocol treatment for any of the above reasons, should be encouraged to remain in the trial for the purpose of providing follow up. All Care Homes that are withdrawn will be included in the data analysis.

<u>Flu vaccination providers (intervention only)</u>

GP Practice and Pharmacy participation in the trial will be voluntary, although providers will be contracted and remunerated for services provided. Should a provider withdraw consent, an alternative provider will be sought and consented and contracted where possible.

6.5 Outcomes

6.5.1 Primary Outcomes

Staff flu vaccination rate is the primary outcome measure and will be calculated as:

Total number of staff vaccinated in a flu season over total number of staff employed at any point throughout that flu season (all directly contracted staff (care staff, cleaners, cooks, administrative staff) + agency staff)

6.5.2 Secondary Outcomes

Staff flu vaccination rate disaggregated by care-giving and non-care giving roles;

staff flu vaccination rate at end of November;

number of staff sick days.

residents' episodes of GP visits, hospitalisations, mortality

6.5.3 Process Evaluation outcomes

Process evaluation outcomes will describe the intervention as delivered in terms of does and fidelity, including adaptations and variations across care homes' :

- To examine and define the mechanisms of action, adaptions and variations across care homes

6.5.4 Health economic outcomes

- Indicative cost estimates of the different intervention elements

6.5.5 Study Within A Trial (SWAT) outcomes

- Measures of care home staff risk-aversion, prosociality and their beliefs about the vaccination behaviour of other staff.

6.5.5 Process Evaluation

[56][57][58], [59][60]A mixed-methods, theory-driven process evaluation will be undertaken in parallel to the FluCare trial. The Theoretical Domains Framework (TDF) (Atkins 2017)) will underpin exploration of the barriers and enablers to flu vaccination uptake within this trial. The process evaluation methods and objectives align with Medical Research Council guidance on evaluating complex interventions (Moore 2013)

6.5.5.1 Process evaluation objectives:

1. To describe the intervention as delivered in terms of dose and fidelity, including adaptations and variations across care homes
2. To explore the effects of individual intervention components on the primary outcomes.
3. To investigate the mechanisms of impact.
4. To describe the perceived effectiveness of relevant intervention components (including videos, leaflets, posters and flu clinics) from participant (care home manager, care home staff and flu clinic providers) perspectives.
5. To describe the characteristics of care homes and participants to assess reach.

6.5.5.2 Process evaluation data collection methods

1. A site profile questionnaire to provide detail on contextual factors:
   All homes will be characterised at the start and end of the trial period to identify characteristics (i.e. home type (private/charity/local authority); size (beds); with/without nursing; number and type of staff; staff working arrangements; infection control policies,

protocols/operating procedures; vaccination policy, guidance/education routinely provided), and changes which may affect intervention implementation during the trial period.

2. Care Home Staff completed - Mechanism of Action Questionnaire (MAQ):
The MAQ comprises of four items, each with a 5-point Likert scale response option (strongly disagree to strongly agree), measuring the extent to which the intervention has addressed the four theoretical domains (figure 1). For each item, participants are also asked to provide an explanation for the response in an extended response option box. Care home staff will complete the MAQ before and after receiving the behaviour change intervention (Figure 1).

   A sample of approximately 20 care homes across both arms of the trial will be invited to distribute the MAQ electronically to all staff at baseline; those responding will be invited to complete the MAQ again at the end of the intervention period. Data will be visually analysed for each respondent to the extent to which the intervention has addressed the barriers to flu vaccination. Variation in MAQ responses between participants, care homes and other contextual factors will be explored further using qualitative interviews.

3. Interviews with purposively selected care home managers and staff, in both arms; pharmacist/healthcare practitioners in the intervention arm:
At the end of the intervention delivery period, we will interview approximately 65 participants: 58 from the intervention arm (12 care home managers 26 care home staff and 20 flu clinic providers) and 7 from the control arm (3 care home managers, 4 care home staff) purposively selecting for type of care home, staff role, characteristics of flu vaccination delivery. A sample of this size will enable representation across the care homes and examination of differences across contexts. Sampling will be supported by, but not limited to, findings from the MAQ to select participants with a range of MAQ outcomes (e.g. participants with most/least barriers addressed), Interview topic guides will ask staff for views on how each BCT was delivered (content); its acceptability, including how compatible it was with routine practices, how each BCT worked within the home (theoretical fidelity), and exploring why BCTs have succeeded in/failed to address certain barriers. Interviews with care home managers will focus on procedures for vaccination clinic visits, staff working arrangements, local infection control policies and other contextual issues affecting intervention delivery. Interviews with pharmacist/healthcare practitioners delivering flu vaccination clinic will elicit experiences of setting up/running flu vaccination visits, and interviews with pharmacists/healthcare practitioners who were unable to deliver on-site clinics will explore the barriers and challenges that prevented them from providing the service. All interviews will be audio-recorded and transcribed verbatim and last no longer than 60 minutes. At this point all identifiers will be removed and transcriptions checked against audio recording. Anonymised transcripts will be uploaded to NViVO for analysis. Audio recordings will be destroyed after analysis and by end of the trial.
For care home managers and staff, demographic information will be collected to include (role in the care home, working hours (part or full-time), age group (under 20, 20-29, 30-39, 40-49, 50-59, 60-69 over 69 years) and ethnic group. A pseudonymised identifier will be used to link demographic data to de-identified transcriptions.

4. Documentary reviews of policies and protocols (e.g. flu campaign policies; infection control procedures, government regulation):
   Relevant protocols, policies and standard operating procedures (e.g. flu campaign policies; infection control procedures) will be reviewed to understand which guidance for flu vaccinations are in place and how they are operationalised within each home, providing context to the analyses.

5. Implementation outcomes:
   In Intervention care homes the dose of intervention material will be described -
   1. No. of times videos played (embedded in videos)
   2. Where posters displayed and engagement with them (interviews)
   3. No. of pharmacy visits to homes (pharmacist log)
   4. Length and time of pharmacist/healthcare practitioner visits to home (Vaccination log)
   5. No of incentive payments made to homes (study records)

Pharmacist/healthcare practitioner running the clinics will use an electronic log (spreadsheet) to report outcomes 3 and 4 contemporaneously. Implementation outcome data will be descriptively analysed.

6.6 Participant Timeline

		Care Home Manager and Staff timelines							
	Enrolment	Allocation	Post-allocation (months)						Post-intervention
TIMEPOINT**	$-t_1$	0	1	2	3	4	5	6	
ENROLMENT:									
Eligibility screen	X								
Informed consent	X								
Allocation		X							
Training: Excel Spreadsheet	X	X							
INTERVENTIONS:									
Arm A (usual care)			←	—	—	—	—	→	
Arm B (behaviour change intervention)			←	—	—	—	—	→	
ASSESSMENTS:									
Care Home profile questionnaire	X							X	
Data spreadsheet completion	X@		X	X	X	X	X	X	
Mechanisms of action questionnaire (+ consent)*	X								X
Understanding Decision Making questionnaire (+consent)**	X								X
Interviews (Managers and staff)*									X
Flu vaccination providers (Pharmacist / GP practice) timelines (Arm B)									
ENROLMENT:									
Eligibility screen	X								
Informed consent	X								
Training: Vaccination Log	X								
ASSESSMENTS:									
Vaccination log completion and send to NCTU			Up to 4 vaccination clinics#						
Interviews*									X

#Intervention care homes only; *procession evaluation care homes only; **homes not participating in process evaluation only, @Data log to be returned prior to randomisation if possible, or as soon as possible thereafter.

6.6.1 Care Home Manager and Staff Assessments

Care Home Manager completed - Site profile questionnaire:

Care home managers will be requested to complete a short survey at the start and again at the end

of the trial period to capture the demographics of the care home including:

- Care home type (i.e., home type (private/charity/local authority);
- Size (number of beds);
- Nursing support (with/without nursing);
- Staffing: number and type of staff and their working arrangements;
- Policies and procedures: Infection control policies, protocols/operating procedures, vaccine policy, guidance/education routinely provided;
- At end of follow-up, any changes which may have affected trial implementation during the trial period.

Care Home Staff completed - MAQ:
Care home staff will be request to complete a short questionnaire at the start and again at the end of the Study (approximately four months apart) to capture key barriers to getting the flu vaccination:

- Whether the care home made it possible for the staff member to get a flu vaccination within regular working hours
- If the care home staff member thinks that getting a flu vaccination has advantages to the people in the care home and their colleagues
- If the care home staff member thinks getting a flu vaccination is consisten with what is expected of their profession
- Whether there are more advantages than disadvantage to the care home staff member getting the flu vaccination.

Care Home Staff completed – Understanding Decision Making (UDM) questionnaire:
Care home staff will be request to complete a short questionnaire at the start and again at the end of the Study (approximately four months apart) to understand decision making. See Appendix 2.

6.6.3 Early Stopping of Follow-up

If a care home manager chooses to stop participation, the home should continue to be followed up as closely as possible to the follow-up schedule defined in the protocol, providing they are willing. They should be encouraged and facilitated not to leave the whole trial, even though they no longer participate in the trial intervention. If, however, the care home manager exercises the view that they no longer wish to be followed up, this view must be respected, and the care home withdrawn entirely from the trial. NCTU should be informed of the withdrawal in writing and will record this on the FluCare database. Data already collected will be kept and included in analyses according to the intention-to-treat principle for all participants who stop follow up early.

Care Homes which withdraw after allocation will not be replaced.

6.6.4 Participant Transfers

We acknowledge that care home staff and residents may move between care homes during the trial. For care home staff last and first date of employment for leavers and new starters, respectively, will be recorded on the data logs. Data on residents living at any time in the care home will contribute to the care home resident data. For the MAQ, and SWAT, following closure of invitation to participate,

new staff starting to work in the care home will not be invited to participate, although vaccination data will still be collected on these employees.

6.6.5 Loss to Follow-up

1. Care home loss to follow-up:

As care homes are being recruited, loss to follow-up is unlikely. However, the study has been powered to accept loss of care homes as a result of closure or sale (20% attrition has been included in the sample size). In the event that a care home has been sold to a new provider, attempts will be sought to obtain informed consent from the new owner/manager. Movement of staff into and away from the care home will be captured during the trial (see below).

2. Staff loss to follow-up:

As part of the staff data log, when staff leave the care home, care home managers are requested to ensured that that the staff stopped working at the home and their vaccination status at that time are logged. An assumed mean number of 54 staff members is used in the sample size, however it is possible that true number will be larger than this. However, the sample size is relatively unchanged by this and does not reduce to less than 90% until the number of staff members reaches 49. In addition, as the study allows for 20% drop-out of care homes the number of staff members can be reduced even further if the drop-out rate is lower.

6.6.6 Trial Closure

The end of the trial is defined as 1 month following the last interview and return of last data collection form, whichever is the latter, to allow for data entry and data cleaning activities to be completed.

6.7 Sample Size

Based on the assumptions that mean (sd) cluster size is 54 staff (25), a coefficient of variation of 0.48 (based on a recent study [61]), control vaccination rate is 55% (assumed higher than the historical rate as COVID has increased interest in vaccination), intervention 75%, intra-cluster correlation coefficient of 0.2 and with 90% power, we require 31 care homes per arm at the two tailed 5% level of significance (62 total). This would also provide 80% power to detect the same difference in the caregiving (non-caregiving) staff subgroup, assumed 40 per care home (14 per care home). We recruit an additional 8 homes per arm to allow for 20% attrition making the final intended sample size of 78 homes in total. The effect of COVID on the control vaccination rate is uncertain. Our sample size also provides over 90% power to detect a difference between a control rate of 40% and intervention rate of 60%. This would still represent a 50% relative increase in vaccination rates than these homes have achieved historically. Such an increase has meaningful benefits for residents since evidence suggests a linear relationship between staff vaccination coverage and resident health [13, 14].[10][11].

6.8 Recruitment, Retention and Data Completeness

6.8.1 Recruitment

UEA School of Economics ethics committee has approved obtaining expressions of interest, prior to consent and recruitment, to participate in the FluCare project. Five approaches to obtaining expressions of interest will be used:

Method 1: contact care homes in England with a flu vaccination rate in the 2021/2022 flu season of <40% identified from the Department of Health and Social Care Capacity Tracker.

Method 2: Place publicity materials in a number of care sector e-newsletters and e-bulletins and via the social media accounts of major care associations (e.g. Care England). The CQC, Care England, Pharmacy chains including Boots UK and Day Lewis Pharmacy, Local authorities, care home chains and care home network organisations (e.g., CHAIN) will also be approached to distribute the publicity material to care homes within England.

Method 3: Members of the trial team will speak at care home sector associations' weekly/monthly care home managers' meetings held online or face to face to publicise the study.

This will include, but not be limited to, National Care Forum's managers' meetings and Care England's regional manager and care home manager meetings.

Method 4: Local Clinical Research Networks will be contacted to circulate the project information to care homes within their region, including those within and external to the ENRICH Network. Members of the research team will also present at CRN meetings to care home managers and staff.

Method 5: Social media campaign, using e.g., Twitter, LinkedIn, FaceBook, WhatsApp

6.8.2 Retention

As the care homes will only be participating in the trial over one winter flu season, we do not anticipate that retention will be an issue.

6.8.3 Data Completeness

Wherever possible mandatory fields will be used increase data completeness. Care Homes and Vaccination Providers will be guided in completing the baseline/monthly staff and resident logs, and the post flu clinic logs, respectively. Where possible, an appropriately completed baseline care home staff log will be required in order for the care home to progress to randomisation. Where this is not possible, the care home should be encouraged to return the log as soon after randomisation as possible. Checks will be carried out by the NCTU operational team to confirm completeness of the log and remdial advice will be provide until an acceptable log has been returned. Care homes and vaccination providers will be reminded to send in data logs in a timely manner. Payment to care homes will be structed according to data collection activity. There will be one payment for completion of all baseline measures completed (including providing staff data logs), 5 payments for return of monthly logs (staff and residents), one per month completed, and a final payment if all 6 monthly logs are completed (staff and residents).

6.9 Assignment of Intervention

6.9.1 Allocation

6.9.1.1 Sequence generation

The sequence will be generated using REDCAP based on stratified randomisation with a binary variable of the percentage of staff identifying as non-white. Blocked randomisation will be undertaken, however given the small number of homes randomised in order to ensure that the risk of imbalance is small a small block size will be used and specified in the allocation system specification.

The classification of the percentage of staff identifying as non-white into two groups will be decided on the basis of the collection of the site profile questionniares which are expected to be collected for a number of care homes prior to the start of any care home being randomised.

6.9.1.2 Allocation concealment mechanism

Randomisation will be undertaken using the REDCAP platform development and managed by NCTU Data Management team. The recruitment and care home facing team will have no access to the allocation sequence.

6.9.1.3 Allocation Implementation

Care homes will be advised as to which arm they have been allocated by a member of the research team.

6.9.2 Blinding

Due to the design of the trial, it is not possible to blind the operational and data management members of the research team. Statistics and Health Economics will be blinded to the randomisation for the purpose of analysis.

6.10 Data Collection, Management and Analysis

6.10.1 Data Collection Methods

Each Care Home will be given a unique trial Participant Identification Number (PID). Data will be collected at the time-points indicated in the Trial Schedule. Each member of staff who logs in to complete the MAQ and or SWAT UDM questionnaire will be allocated a unique ID linked to their care home.

Vaccination logs, Care Home Spreadsheets, and Resident logs will be provided in a simple to use format either in paper or Excel spreadsheet. Care homes will be provided with instructions of how to pseudonymise the spreadsheet ahead of sending to NCTU for collating into the trial database. Vaccination logs will not contain names of care home staff. Vaccination providers will be requested to complete a log of care home staff attending the clinic and leave this with the care home manager for their records.

For the MAQ, care home staff will be required to use the online platform (REDCap) to complete an online consent form prior to completing the questionnaire.

Data collection, data entry and queries raised by a member of the FluCare trial team will be conducted in line with the NCTU and trial specific Data Management Standard Operating Procedures.

Identification logs, screening logs and enrolment logs will be kept electronically in a password protected secure environment.

Logs containing care home manager contact information will be stored on a spreadsheet on UEA's secure server to enable care home managers to be contacted by site staff, and the central trial team for the purpose of sending newsletters during the trial. There will be a clear logical separation of care home identifiable data from the trial data.

Resident aggregate data will be provided by care home managers using an excel spreadsheet or via the REDCap platform. Aggregate data will be tracked by care home number. No individualised resident data will be collected.

For Department of Health and Social Care (DHSC) Capacity Tracker data, care home identifiers will be submitted to DHSC by NCTU Data Management for matching with the Capacity Tracker data. Aggregate flu vaccination data on permanent staff, agency workers and volunteers, and residents, within the participating care homes will be sent securely to NCTU. As the Capacity Tracker only holds aggregate data, there is no risk of sharing of personal identifiable data. A data sharing agreement will be in place between the DHSC and Sponsor prior to the release of the data.

Clinical trial team members will receive trial protocol training. All data will be handled in accordance with the Data Protection Act 2018.

For the SWAT, care home staff will be required to use Qualtics, a secure, web-based software platform to provide consent to participate and retain identifiable data for the purpose of sending a voucher, and follow-up questionnaire. The UDM questionnaire (the incentivised decision-making portion of the study) will be hosted on O-Tree, a package built on top of the widely-used Django platform for building and deploying websites, to produce a bespoke interface. The O-Tree application will be hosted on a server located in the UK/EU. A per participant unique link to the O-Tree application from Qualtrics will be gernated by creating an anonymous, opaque identifier in Qualtrics and recording this in the responses in the O-Tree application; all data recorded in the O-Tree application will be fully anonymous.

6.10.2 Data Management

Data will be entered under the care home number and participant PID number onto the central database stored on the servers based at NCTU. Access to the database will be via unique, individually assigned (i.e., not generic) usernames and passwords, and only accessible to members of the FluCare trial team, and external regulators if requested. The servers are protected by firewalls and are patched and maintained according to best practice. The physical location of the servers is protected physically

and environmentally in accordance with University of East Anglia's General Information Security Policy 3 (GISP3: Physical and environmental security).

The database and associated code have been developed by NCTU Data Management, in conjunction with the FluCare trial team. The database software provides a number of features to help maintain data quality, including; maintaining an audit trail, allowing custom validations on all data, allowing users to raise data query requests, and search facilities to identify validation failure/ missing data.

After completion of the trial, the database will be retained on the servers of NCTU for on-going analysis.

Participant identifiable data will be held within the REDCap database separated from the research data by logical separation. Identifiable data will be deleted at the end of the study, with the exception of information required for financial regulators (for payment of vouchers).

For the SWAT, the data will be entered under the care home number and participant PID number onto the Qualtics and central database in the UK/EU. This will be overseen by the Laboratory for Economic and Decision Research at UEA.

6.10.3 Non-Adherence and Non-Retention

Non-adherence to the allocated trial arm and withdrawal of consent will be captured in trial logs and reviewed by the Programme Management Group. These data will be reviewed as part of the progression criteria to the definitive trial.

6.10.4 Statistical Methods

Analysis based on the intention-to-treat principle, using all available data. The difference in vaccination rates will be presented for each group separately and compared using a random effect logistic regression model at the staff-level. The random effect will be the home. If staff data are missing, then the results' sensitivity will be assessed by imputation with two strategies: missing data will be assumed to be not vaccinated; multiple imputation will be attempted using iteratively chained equations. Given the amount of data to base the imputation model on, the primary analysis will remain the observed data analysis. Secondary outcomes will also be compared using random effect models. Assumptions will be checked and if violated then either a nonparametric bootstrap or cluster-summary approach will be used. The analysis will consider firstly, all staff, then all caregiver and non-care giver staff groups separately. A subgroup analysis will investigate if there is a differential effect in the ethnic minorities staff group. We shall also examine for subgroup effect from selected characteristics of the care home using SPQ and average MAQ responses. Full details will be agreed and documented in the Statistical Analysis Plan (SAP) before final analysis. Where there is a discrepancy between the SAP and protocol, the SAP will have priority.

6.10.4.1 SWAT Statistical Methods

We will estimate OLS regressions where the dependent variable is vaccination status and the right-hand side variables of interest will be interactions between whether a staff member comes from a care home which received the intervention and each decision-making characteristic (i.e. risk aversion, prosociality or beliefs). A positive significant coefficient on such an interaction would mean

that the intervention is more likely to work (i.e. lead to vaccination) on staff with a higher degree of the decision-making characteristic (e.g. risk-aversion).

Given how busy care home staff are, we anticipate significant selection amongst those staff who respond to our survey. However, we have considerable staff-level data on all employees of the 58 care homes in the FluCare trial dataset (approximately 2900 staff = 58 care homes, each with approximately 50 staff). We will thus examine test whether respondents differ significantly from the population of the care home staff in the trial based on observable characteristics (e.g. sociodemographics, number of sick days etc) and use existing literature to inform whether any biases are implied by the selection observed.

A data analysis plan will be written and published prior to data analysis.

6.10.5 Health Economic Methods

We will conduct a within trial cost-consequences analysis (CCA) comparing costs and outcomes between trial arms across different perspectives/stakeholders (e.g., care homes, NHS and staff). CCA is a standard evaluation approach recognized as being particularly useful for evaluating interventions that have impacts on multiple domains of outcome and perspectives [[62][63].

We will determine the resources involved in, and associated costs of, delivering the FluCare intervention. Resources required for intervention delivery are expected to consist primarily of clinician time to deliver the FluCare clinics and the vaccinations. Information on these and other resources will be collected from clinic logs, process evaluation and augmented with expert opinion as need. Resources will be costed in the most recent cost year for which published NHS and PSS unit costs (e.g. [64]) are available.

If the intervention is effective, we will determine the cost per increased percentage point of vaccination rate. We will also consider impacts on care home staff [staff sickness and agency staff utilisation (restricted to those homes that can use such staff)] and resident health (rates of: GP visits; nurse visits; hospitalisation; all-cause mortality). Secondary analysis will disaggregate appropriate results by staff type (e.g., care home versus agency) and role. We will explore if the interventions costs may be offset by reduction in the use of other resources (e.g., fewer resident GP visits). Additionally, we will also explore crude valuations of life years gained (for example, noting any differences in resident mortality multiplied by typical survival periods).

The analysis will adopt a ‘within trial’ approach, i.e., up to the six months of the trial. Given the duration of less than a year, discounting will not be required. In line with the statistical analysis, we will analyse patterns of missing data, and where appropriate, multiple imputation will be used to impute data. Decisions relating to the treatment of missing data will be made in consultation with the study CIs and statistician. If data is imputed, then results will be presented for both the imputed data as well as a complete case analysis (CCA). Data will be analysed on an intention-to-treat basis. If adjustment for other factors is needed (e.g., care home size), costs and effects will be analysed using appropriate regression-based methods. Analyses will be performed in a variety of packages, likely to include: MS Excel; R; and STATA.

In accordance with NCTU practice we will draft a health economic analysis plan (HEAP) prior to conducting the economic analysis. This will be shared and discussed with members of the TMG and other key personnel before analysis is undertaken.

6.10.6 Process Evaluation Methods

Hypothetical propositions from the feasibility study of how BCTs enhance reach and fidelity of the intervention will provide the analysis structure for process evaluation main trial data.

6.10.6.1 Analysis of implementation outcomes

The primary aim of this analysis will be to summarise the amount (or dose) of the intervention received in each intervention home. The outcomes will be summarised the outcomes using tabulations and descriptive statistics. The data will potentially be included in three potential analyses:

a) The association between the effectiveness outcomes and the implementation outcomes will be assessed at the home level in the intervention arm by correlating the change in the effectiveness outcome and the implementation outcome;
b) Each home will be classified by total amount of 'dose' implemented and a test-for-trend will be undertaken, including the control homes with zero dose, to assess if the amount of the intervention received is associated with the outcome;
c) We will attempt to include the implementation outcomes in the mediation analysis described in the MAQ analysis below.

6.10.6.2 Analysis of MAQ data

We will analyse MAQ data at the home level to describe average intervention effects on the determinants of vaccination (Figure 1) at each site. Additionally, an analysis of each of the five BCTs (Figure 1) will be undertaken to determine which of the five contribute to outcome changes. Potential mediation effect from the MAQ will be investigated if there is both a significant effect of the intervention and a significant change in MAQ from baseline to outcome in either the control or intervention arms. Firstly, a scatter-plot of the change in MAQ response versus the care-home vaccination rate will be conducted for each response to MAQ separately. Secondly, a direct acyclie graph (DAG) model will be developed for each MAQ question separately. This model will allow the estimation of the direct effect of the intervention and the indirect effect via the change in MAQ. As it is possible that the mediators act in a non-independent fashion a multiple mediation analysis will be considered, but may be limited due to the number of care homes.

6.10.6.3 Analysis of interviews

We will draw on Braun and Clarke's thematic analysis as a 'contextualist' method [ref], to inductively analyse interview transcripts, examining how themes map onto BCTs at different contextual levels. We will analyse interview data using two methods. Firstly drawing on Braun and Clarke's thematic analysis as a 'contextualist' method [66], we will inductively analyse interview transcripts. We will follow the 6 steps of thematic analysis outlined in Braun and Clarkes early work, remaining aware of the importance of acknowledging and reporting the subjectivities of the researchers and the ways in

which meaning are iteratively developed rather than prosaically ‘found’ [67]. Following the thematic analysis we will Explore the data through the behavioural change method of deductively map codes to the TDF. This dual approach will enable understanding of meanings and actions within social contexts and through individual behaviours; it will support triangulation of findings. Interview transcripts will be analysed using NVivo software. For intervention arm participants, we will evaluate how the process and content of the intervention functioned from the participants’ perspective, identifying how much barriers were overcome by different BCTs to increase vaccination rates and intervention sustainability over time. First order codes will be analysed to develop higher order codes to better facilitate understanding of emerging relationships between implementation, BCTs and outcomes. The analysis team will include four researchers experienced in qualitative research methods and two with a speciality in using the TDF. They each bring differing perspectives and discussions within the group will support the trustworthiness of results.

Any theme not readily mapping onto a theoretical domain will be discussed and assigned to a domain or an additional domain added. We will then triangulate the themes with MAQ and implementation outcome findings, “following a thread” [65] to test and explore emerging interpretations between different BCTs and outcomes. This will enable us to produce explanations for the main trial findings and preliminary recommendations for wide-scale implementation. Throughout analysis, project team members and Lived Advisory Group will review findings, discuss emerging interpretations and final explanations for the main trial effects.

6.11 Data Monitoring

6.11.1 Data Monitoring Committee (DMC)

The intervention being evaluated is to encourage and support individuals to access flu vaccination. This trial is not designed to evaluate the safety of the flu vaccine. As such, the DMC and PSC have agreed that there are no safety issues. The primary risk to the project are trial failure (for example failure to recruit and poor data collection). Further details of the roles and responsibilities of the DMC, including membership, relationships with other committees, decision making processes, and the timing and frequency of interim analyses (and description of stopping rules and/or guidelines where applicable) are described in detail in the FluCare DMC Terms of Reference (ToR).

6.11.2 Interim Analyses

No interim analyses are planned.

6.11.4 Quality Assurance and Control

6.11.4.1 Risk Assessment

The Quality Assurance (QA) and Quality Control (QC) considerations for the FluCare trial are based on the standard NCTU Quality Management Policy that includes a formal Risk Assessment, and that acknowledges the risks associated with the conduct of the trial and proposals of how to mitigate them through appropriate QA and QC processes. Key risks identified in this project include recruitment (care homes and vaccination providers), intervention production and delivery, funding (specifically excess treatment costs) and data collection (staff, resident and vaccination logs). The risks will be detailed in a risk assessment apperovided by the PMG prior to the start of the project.

QA is defined as all the planned and systematic actions established to ensure the trial is performed and data generated, documented and/or recorded and reported in compliance with the principles of GCP and applicable regulatory requirements. QC is defined as the operational techniques and activities performed within the QA system to verify that the requirements for quality of the trial related activities are fulfilled. The trial is embedded within the NCTU Quality Management System, and NCTU working practices and working instructions will be followed throughout trial set-up, delivery and analysis. QC checks will be performed on consent, data collection and Quality Management and Monitoring Plan will be produced for this trial. This will include QC checks on consent, intervention initiation (receipt of intervention materials by sites) and data collection (frequency and quality).

6.11.4.2 Central Monitoring at NCTU

Delegated FluCare trial team members will review data for errors and missing key data points. The trial database will also be programmed to generate reports on errors and error rates. Essential trial issues, events and outputs, including defined key data points, will be detailed in the FluCare trial Data Management Plan.

6.11.4.3 On-site Monitoring

Due to the single centre recruiting design and the low-risk nature of the trial, onsite monitoring will not be undertaken. As NCTU are involved in all elements of the project at the single centre (UEA) any issues that arise will be escalated accordingly.

6.11.4.4 Trial Oversight

Trial oversight is intended to preserve the integrity of the trial by independently verifying a variety of processes and prompting corrective action where necessary. The processes reviewed relate to participant enrolment, consent, eligibility, and allocation to trial groups; adherence to trial interventions and policies to protect participants, including reporting of harms; completeness, accuracy and timeliness of data collection; and will verify adherence to applicable policies detailed in the Compliance section of the protocol. Independent trial oversight complies with the NCTU trial oversight policy.

6.11.4.4.2 Programme Management Group

A Programme Management Group (PMG) will be set up to assist with developing the design, co-ordination, and day to day operational issues in the management of the trial, including budget management, and strategic management of the trial. The membership includes the co-Chief Investigators (Behavorial Economist and Pharmacist/Clinical Trialist); co-investigators with expertise in trial operations, PPI engagement, public health, process evaluation, qualitative research and behavioural science, health economics statistics and intervention evaluation, advisors on configuring and commissioning pharmacy services and implementation, PPI including care home management and relatives of care home resident), and research and NCTU staff supporting care home research delivery, process evaluation, and trial set-up and delivery. A sub-group of the PMG meet weekly to review, agree and implement deliverables, and full meetings held approximately quarterly to review progress oversee trial conduct . The authority will be covered in the PMG terms of reference.

6.11.4.4.3 Independent Programme Steering Committee

The Independent Programme Steering Committee (PSC) is the independent group responsible for oversight of the trial in order to safeguard the interests of trial participants. The PSC provides advice

to the CI, NCTU, the funder and sponsor on all aspects of the trial through its independent Chair. The independent membership includes Statistician, Public Health Specialist, Trialist, Behavioural Scientist, three stakeholder representatives (Care England; National Care Forum and Pharmacy Chain) and two PPI members. The PSC meets approximately 6 monthly to review progress, including mitigations as necessary. Authority of the PSC is covered in the PSC terms of reference.

In this propject, the Data Management Committee (DMC) will meeting jointly with the PSC.

6.11.4.4.4 Data Monitoring Committee

The Data Monitoring Committee (DMC) has been appointed to ensure additional rigour of the trial. As the intervention is to improve care home staff access to flu vaccination, and not the safety of the flu vaccination, there are no participant safegurarding issues. As care home managers are responsible for data collection and will be required to submit monthly staff and resident data logs during the trial for monitoring purposes, but will not be uploaded to the database until the end of the follow-up period, the DMC will not have access to unblinded accumulating comparative data. The DMC will meet jointly with the Programme Steering Commmittee during the trial to review trial progresss including recruitment and data log return. The IDMC will also consider data in accordance with the statistical analysis plan and will advise the TSC through its Chair.

6.11.4.4.5 Trial Sponsor

The University of East Anglia is the trial sponsor. The role of the sponsor is to take on responsibility for securing the arrangements to initiate, manage and finance the trial. The Sponsor is responsible for ensuring that the study meets the relevant standards and makes sure that arrangements are put and kept in place for management, monitoring and reporting. The University of East Anglia has delegated some Sponsor’s activities to the CI and NCTU, these are documented in the Collaboration Agreement.

7 Ethics and Dissemination

7.1 Research Ethics and Health Research Authority Approval

Before initiation of the trial at any clinical site, the protocol, all informed consent forms and any material to be given to the prospective participant will be submitted to the NIHR Regulatory Ethics Committee (REC) and to the HRA for approval. Any subsequent amendments to these documents will be submitted for further approval.

The rights of the participant to refuse to participate in the trial without giving a reason must be respected.

7.2 Competent Authority Approvals

This is not a Clinical Trial of an Investigational Medicinal Product (IMP) as defined by the EU Directive 2001/20/EC. Therefore, a CTA is not required in the UK.

7.3 Other Approvals

Confirmation from the care home, GP or pharmacy will take the form of a site agreement signed by the Sponsor and the relevant care home.

The protocol has received formal approval and methodological, statistical, clinical and operational input from the NCTU Protocol Review Committee.

7.4 Amendments

Amendments to the Protocol and other documents (e.g., changes to eligibility criteria, outcomes, sample size calculations, analyses) will be agreed by the PMG. Such amendments will be forwarded to the Sponsor for confirmation as to whether it is either substantial or non-substantial and will then be submitted to the Health Research Authority or Ethics Committee for categorisation and approval. Once the amendment has been categorised it will be sent to recruiting site for implementation in accordance with standard HRA processes and timescales. Amendments must not be implemented until HRA approval is received and recruiting site has confirmed acceptance. Notification will be sent by NCTU to trial personnel to confirm when an amendment can be implemented.

7.5 Consent

Care Homes

Care home managers / owners will be required to give informed consent for their care home to participate in the trial. Care home managers or owners will be provided with a Participant Information Sheet (PIS) and given time to read it fully. Following a discussion with a qualified investigator or suitable trained and authorised delegate, any questions will be satisfactorily answered and if the participant is willing to participate, informed electronic consent (e-consent)will be obtained. During the consent process it will be made completely and unambiguously clear that the participant is free to refuse to participate in all or any aspect of the trial, at any time and for any reason, without incurring any penalty or affecting their rights.

GP / Pharmacist

Potential vaccination providers will be initially identified from the care homes. GP practices and local pharmacies will be approached to establish willingness to participate in the trial and provide vaccination services to the care homes. Once interest in the study has been established, e-consent will be sought following the same procedure outlined above.

Care Home Staff (MAQ)

Care home staff will be invited to participate in completion of the MAQ by electronic means (i.e., email, text, whatsapp) via their care home manager. The invitation will include a link to direct the member of staff to an online form which will include the PIS detailing the study, what they will be doing, and how their contact information will be used.

This will then progress to the ICF which once completed will take them directly to the MAQ questionnaire. Once a member of staff provides e-consent, they will be emailed a copy of the PIS and consent form. If staff no longer want to participate they can either contact the research team or not respond to requests to complete the second MAQ. The first 500 care home staff to complete both MAQs will be given a £10 thank you voucher.

Care Home Staff (UDM Questionnaire)

Care home staff will be invited to participate in completion of the UDM questionnaire for the Study Within a Trial by electronic means (i.e., email, text, whatsapp) via their care home manager. The

invitation will include a link to direct the member of staff to an online form which will include the PIS detailing the study, what they will be doing, and how their contact information will be used.

This will then progress to the ICF which once completed will take them directly to the SWAT questionnaire. Once a member of staff provides e-consent, they will be emailed a copy of the PIS and consent form. Following completion of the first questionnaire, if staff no longer want to participate they can either contact the research team or not respond to requests to complete the second SWAT Questionnaire. As a thank you, care home staff will receive payment (between £5 and £14) for responding to the survey in Amazon vouchers. The exact value of Amazon vouchers received will depend on their responses to questions in the survey, however we expect each respondent to receive approximately £10 per survey.

Consent to participate in interviews

For care home managers, participation in process evaluation interviews is a requirement of participation in the study. For care home staff, care home managers will be asked to raise awareness of the interviews among care home staff though posters with research contact details, or electronically providing care home staff with a brief summary of reasons for interviews and researchers contact details to express interest. Once the study team receive an expression of interest, they will send the staff member the PIS and consent form. They will follow up no sooner than 48 hours later to see if they wish to take part. If so researchers will answer any further questions agree a date and time for a virtual interview and ask them to sign the e-consent form prior to the interview.

Pharmacists/healthcare professional in the intervention arm will be invited to participate in interviews. As multiple pharmacists/healthcare professionals may be involved in delivery of the flu clinics, the lead pharmacist will be requested to distribute PIS and consent forms to colleagues who have delivered flu clinics for the colleagues to confirm they are willing to participate in the interview.

Care Home managers and lead pharmacists at the participating site not selected to participate in the interviews will be sent a letter advising them of the outcome.

Copies of the approved consent forms are available from the NCTU trial team.

7.6 Confidentiality

Any paper copies of personal trial data will be kept at the participating site in a secure location with restricted access. Following consent, identifiable data will be kept on the trial database to allow authorised members of the trial team to contact care home staff for follow-up assessments. Only authorised trial team members will have password access to this part of the database. This information will be securely destroyed within 6 months of the end of the trial, expect for where required to be retained to meet financial regulations.

Confidentiality of care home staff personal data is ensured by not collecting names on CRFs and limiting access to personal information held on the database at NCTU. At trial enrolment the member of staff will be issued a participant identification number, and this will be the primary identifier for the participant. Care Home Manager and Pharmacy or GP Practice Consent will be collected electronically

following discussion with the research team. For the MAQ, Care Home Staff consent will also be collected electronically, and staff email addresses will be retained to facilitate sending of follow-up questionnaires. Identifiable data will be held securely with logical separation from outcome data. Identifiable data will be deleted within 6 months of study completion.

7.7 Declaration of Interests

The investigators named on the protocol have no financial or other competing interests that impact on their responsibilities towards the scientific value or potential publishing activities associated with the trial.

7.8 Indemnity

As sponsor, UEA has appropriate indemnity to cover their responsibilities as Sponsor and any liability in respect of this. UEA holds insurance to cover participants for injury caused by their participation in the study. Participants may be able to claim compensation if they can prove that UEA has been negligent. However, as this study is being carried out in a care home, the care home continues to have a duty of care to the participant in the study; UEA does not accept liability for any breach in the care home's duty of care (to staff or resident), or any negligence on the part of care home employees. This does not affect the participant's right to seek compensation via the non-negligence route.

7.9 Finance

FluCare is fully funded by an NIHR PHR grant number NIHR133455. It is not expected that any further external funding will be sought.

7.10 Archiving

The investigators agree to archive and/or arrange for secure storage of FluCare trial materials and records, including consent forms for 10 years after the close of the trial unless otherwise advised by the NCTU. The pseudonymisation key will only be stored with the care home manager; there is no intention to undertake additional linkage after the study has ended.

7.11 Access to Data

Requests for access to trial data will be considered, and approved in writing where appropriate, after formal application to the Programme Management Group and Programme Steering Committee. Considerations for approving access are documented in the PMG/TSC Terms of Reference. In line with NIHR desire for data to be shared wherever possible, we will endeavour to facilitate the request following appropriate review by sponsor and research team.

7.12 Ancillary and Post-trial Care

The Sponsor is not responsible for providing ancillary or post trial care following influenza vaccination advocated by this trial. Should care home staff decide to receive the influenza vaccination, any issues arising from that vaccination should be reported to MHRA using the standard yellow card reporting process

7.13 Publication Policy

7.13.1 Trial Results

The results of the trial will be disseminated regardless of the direction of effect. Authorship guidelines have been agreed as part of the overarching research programme (see document FluCare Publication Policy). Following publication of the definitive trial, data will be made available from both the feasibility and definitive trials for secondary research purposes.

A protocol paper will be published for FluCare Work Package 3.

8 Protocol Amendments

Protocol Version	Date	Summary of Changes
V1.0		Pre Ethical/HRA approval
V1.1	05/08/22	Original
V1.2	15/11/22	Minor text clarifications and edits<br><br>Clarification that data logs will be collected prior to randomisation or as near to randomisation date as possible.
V1.3	10/01/23	Minor text clarification and edits<br><br>Clarification of the demographics of care home staff interview participants.
V1.4	27/02/23	Edits and additions to sections 6.5.5.2 & 7.5 to allow the Process Evaluation team to invite all Pharmacists/Healthcare Professionals in the intervention arm to be interviewed. To include those who were unable to deliver on-site flu staff clinics to explore the barriers and challenges that prevented them from providing the service.<br><br>Minor typographical errors

9 References

[1] "Chan AW, Tetzlaff JM, Altman DG et Al. SPIRIT 2013 Statement: Defining Protocol Items for Clinical Trials. Ann Intern Med 2013; 158:200-207."

[2] A. W. Chan *et al.*, "SPIRIT 2013 explanation and elaboration: guidance for protocols of clinical trials," *BMJ*, vol. 346, Jan. 2013, doi: 10.1136/BMJ.E7586.

[3] P. H. England, "Surveillance of influenza and other respiratory viruses in the United Kingdom : Winter 2018 / 19 About Public Health England," 2019.

[4] L. E. Lansbury, C. S. Brown, and J. S. Nguyen-Van-Tam, "Influenza in long-term care facilities," *Influenza Other Respi. Viruses*, vol. 11, no. 5, pp. 356–366, 2017, doi: 10.1111/irv.12464.

[5] G. Matias, R. J. Taylor, F. Haguinet, C. Schuck-Paim, R. L. Lustig, and D. M. Fleming, "Modelling estimates of age-specific influenza-related hospitalisation and mortality in the United Kingdom," *BMC Public Health*, vol. 16, no. 1, pp. 1–9, 2016, doi: 10.1186/s12889-016-3128-4.

[6] A. C. Hayward *et al.*, "Effectiveness of an influenza vaccine programme for care home staff to prevent death, morbidity, and health service use among residents: Cluster randomised controlled trial," *Br. Med. J.*, vol. 333, no. 7581, pp. 1241–1244, 2006, doi: 10.1136/bmj.39010.581354.55.

[7] A. C. Hayward, "Influenza vaccination of healthcare workers is an important approach for reducing transmission of influenza from staff to vulnerable patients," *PLoS One*, vol. 12, no. 1, pp. 1–5, 2017, doi: 10.1371/journal.pone.0169023.

[8] J. Campbell, "Influenza vaccination for healthcare workers who care for people aged 60 or older living in long-term care institutions," *Int. J. Nurs. Pract.*, vol. 25, no. 3, 2016, doi: 10.1111/ijn.12730.

[9] M. Lemaitre *et al.*, "Effect of influenza vaccination of nursing home staff on mortality of residents: A cluster-randomized trial," *J. Am. Geriatr. Soc.*, vol. 57, no. 9, pp. 1580–1586, 2009, doi: 10.1111/j.1532-5415.2009.02402.x.

[10] A. M. Wendelboe, C. Grafe, M. McCumber, and M. P. Anderson, "Inducing Herd Immunity against Seasonal Influenza in Long-Term Care Facilities through Employee Vaccination Coverage: A Transmission Dynamics Model," *Comput. Math. Methods Med.*, vol. 2015, 2015, doi: 10.1155/2015/178247.

[11] C. Van Den Dool, M. J. M. Bonten, E. Hak, J. C. M. Heijne, and J. Wallinga, "The effects of influenza vaccination of health care workers in nursing homes: Insights from a mathematical model," *PLoS Med.*, vol. 5, no. 10, pp. 1453–1460, 2008, doi: 10.1371/journal.pmed.0050200.

[12] A. N. M. Ng and C. K. Y. Lai, "Effectiveness of seasonal influenza vaccination in healthcare workers: A systematic review," *J. Hosp. Infect.*, vol. 79, no. 4, pp. 279–286, 2011, doi: 10.1016/j.jhin.2011.08.004.

[13] M. Pereira, S. Williams, L. Restrick, P. Cullinan, and N. S. Hopkinson, "Healthcare worker influenza vaccination and sickness absence – An ecological study," *Clin. Med. J. R. Coll. Physicians London*, vol. 17, no. 6, pp. 484–489, 2017, doi: 10.7861/clinmedicine.17-6-484.

[14] WHO, "Continuity and coordination of care: a practice," 2018.

[15] B. Franklin and D. Hochlaf, “An Economic Analysis of Flu Vaccination,” no. July, 2018.

[16] WHO, “Prevention and control of influenza pandemics and annual epidemics,” *Fifty-sixth World Heal. Assem.*, no. May, pp. 38–40, 2003.

[17] Public Health England (PHE), “Seasonal influenza vaccine uptake in healthcare workers (HCWs) in England: winter season 2019 to 2020,” no. February, pp. 1–25, 2020.

[18] T. K. Burki, “Concerns over low uptake of flu vaccination in social care,” *The Lancet. Respiratory medicine*, vol. 6, no. 12. NLM (Medline), p. 898, Dec. 01, 2018, doi: 10.1016/S2213-2600(18)30471-5.

[19] et al. Patel, A., “Survey of frontline workers in social care settings,” 2020.

[20] “NHS Capacity Tracker dataset, NHS England and NHS Improvement,” 2021.

[21] NICE, “Flu vaccination: Increasing uptake, National Institute for Clinical Excellence, Editor. 2018.”

[22] P. H. England, “Flu and flu vaccination 2019/20: A toolkit for care homes. 2019.”

[23] “NHS England and NHS Improvement, Social care: guidance for workforce flu vaccination, NHS England and NHS Improvement, Editor. 2019.”

[24] “Newey, S., Expect flu to surge next winter as lockdown has led to low levels, experts warn, in The Telegraph. 2021.”

[25] “Lazarus, R., et al.,, ComFluCOV Study Protocol, University Hospitals Bristol and NHS Foundation Trust, Editor. 2021.”

[26] M. R. Council., “Developing and evaluating complex interventions. 2006.”

[27] L. Atkins *et al.*, “A guide to using the Theoretical Domains Framework of behaviour change to investigate implementation problems,” pp. 1–18, 2017, doi: 10.1186/s13012-017-0605-9.

[28] “Bechini, A., et al., Utility of Healthcare System-Based Interventions in Improving the Uptake of Influenza Vaccination in Healthcare Workers at Long-Term Care Facilities: A Systematic Review. Vaccines, 2020. 8(2): p. 165.”

[29] “Jenkin, D.C., et al., A rapid evidence appraisal of influenza vaccination in health workers: An important policy in an area of imperfect evidence. Vaccine: X, 2019. 2: p. 100036.”

[30] “Lytras, T., et al., Interventions to increase seasonal influenza vaccine coverage in healthcare workers: A systematic review and meta-regression analysis. Human Vaccines & Immunotherapeutics, 2016. 12(3): p. 671-681.”

[31] “Local Government Association, Increasing uptake for vaccinations: Maximising the role of councils, Local Government Association, Editor. 2020.”

[32] “Sand, K.L., et al., Increasing Influenza Immunization for Long-Term Care Facility Staff Using Quality Improvement. Journal of the American Geriatrics Society, 2007. 55(11): p. 1741-1747.”

[33] “World Health Organization, International Clinical Trials Registry Platform (ICTRP).”

[34] “World Health Organization, Report of the Sage Working Group on Vaccine Hesitancy. 2014,

World Health Organization."

[35] "Booth, R., Why do UK care homes employ so many temporary staff?, in The Guardian. 2020."

[36] "Johnston, S.S., et al., Employees' willingness to pay to prevent influenza. Am J Manag Care, 2010. 16(8): p. e205-14."

[37] "Kenny, E., et al., Barriers to seasonal influenza vaccine uptake among health care workers in long- term care facilities: A cross-sectional analysis. British Journal of Health Psychology, 2020. 25(3): p. 519-539."

[38] "Halpin, C. and B. Reid, Attitudes and beliefs of healthcare workers about influenza vaccination. Nursing Older People, 2019. 31(2): p. 32-39."

[39] "Boey, L., et al., Attitudes, believes, determinants and organisational barriers behind the low seasonal influenza vaccination uptake in healthcare workers – A cross-sectional survey. Vaccine, 2018. 36(23): p. 3351-3358."

[40] "Elias, C., et al., Seasonal influenza vaccination coverage and its determinants among nursing homes personnel in western France. BMC Public Health, 2017. 17(1)."

[41] "Cane, J., et al., From lists of behaviour change techniques (BCTs) to structured hierarchies: Comparison of two methods of developing a hierarchy of BCTs. British Journal of Health Psychology, 2015. 20(1): p. 130-150."

[42] "Scott, S., et al., A practitioner behaviour change intervention for deprescribing in the hospital setting. Age and Ageing, 2020."

[43] "Michie, S., L. Atkins, and R. West, The APEASE criteria for designing and evaluating interventions. The behaviour change wheel: a guide to designing interventions. London: Silverback Publishing, 2014."

[44] "Razai, M.S., et al., Covid-19 vaccine hesitancy among ethnic minority groups. BMJ, 2021: p. n513."

[45] "Michie, S., M.M. Van Stralen, and R. West, The behaviour change wheel: A new method for characterising and designing behaviour change interventions. Implementation Science, 2011. 6(1): p. 42."

[46] "Shroufi, A., et al., Influenza vaccine uptake among staff in care homes in Nottinghamshire: A random cluster sample survey. Public Health, 2009. 123(10): p. 645-649."

[47] "Rebmann, T., et al., Seasonal influenza vaccine compliance among hospital-based and nonhospital- based healthcare workers. infection control and hospital epidemiology, 2012. 33(3): p. 243-249."

[48] "Mounier-Jack, S., et al., Organisational factors affecting performance in delivering influenza vaccination to staff in NHS Acute Hospital Trusts in England: A qualitative study. Vaccine, 2020. 38(15): p. 3079-3085."

[49] "Allan, S. and J.E. Forder, Care markets in England: Lessons from research. 2012."

[50] "Torjesen, I., Flu vaccine shortages: NHS England must improve planning to avoid a repeat of this year's delays. BMJ, 2018. 363: p. k4547."

[51] “Apenteng, B.A. and S.T. Opoku, Employee influenza vaccination in residential care facilities. American Journal of Infection Control, 2014. 42(3): p. 294-299.”

[52] “Yue, X., et al., Workplace Interventions and Vaccination-Related Attitudes Associated With Influenza Vaccination Coverage Among Healthcare Personnel Working in Long-Term Care Facilities, 2015–2016 Influenza Season. Journal of the American Medical Director.”

[53] “Borgey, F., et al., Effectiveness of an intervention campaign on influenza vaccination of professionals in nursing homes: A cluster-randomized controlled trial. Vaccine, 2019. 37(10): p. 1260-1265.”

[54] “Nace, D.A., et al., Impact of the Raising Immunizations Safely and Effectively (RISE) Program on Healthcare Worker Influenza Immunization Rates in Long Term Care Settings. Journal of the American Medical Directors Association, 2012. 13(9): p. 806-810.”

[55] “Kimura, A.C., et al., The Effectiveness of Vaccine Day and Educational Interventions on Influenza Vaccine Coverage Among Health Care Workers at Long-Term Care Facilities. American Journal of Public Health, 2007. 97(4): p. 684-690.”

[56] G. F. Moore *et al.*, “Process evaluation of complex interventions: Medical Research Council guidance,” *BMJ*, vol. 350, 2015, doi: 10.1136/bmj.h1258.

[57] P. Craig, P. Dieppe, S. Macintyre, S. Mitchie, I. Nazareth, and M. Petticrew, “Developing and evaluating complex interventions: The new Medical Research Council guidance,” *Bmj*, vol. 337, no. 7676, pp. 979–983, 2008, doi: 10.1136/bmj.a1655.

[58] P. Hawe, A. Shiell, and T. Riley, “Theorising interventions as events in systems,” *Am. J. Community Psychol.*, vol. 43, no. 3–4, pp. 267–276, 2009, doi: 10.1007/s10464-009-9229-9.

[59] “Moore, G.F., et al., From complex social interventions to interventions in complex social systems: Future directions and unresolved questions for intervention development and evaluation. Evaluation, 2018. 25(1): p. 23-45.”

[60] “Murdoch, J., Process evaluation for complex interventions in health services research: analysing context, text trajectories and disruptions. BMC Health Services Research, 2016. 16(1): p. 407.”

[61] P. A. Logan *et al.*, “A multidomain decision support tool to prevent falls in older people: the FinCH cluster RCT,” *Health Technol. Assess.*, vol. 26, no. 9, pp. 1–136, Jan. 2022, doi: 10.3310/CWIB0236.

[62] “Methods for the development of NICE public health guidance (third edition) Process and methods,” 2012, Accessed: Jun. 08, 2022. [Online]. Available: www.nice.org.uk/process/pmg4.

[63] R. Hunter and J. Shearer, “Cost-consequences analysis - an underused method of economic evaluation,” *Natl. Inst. Heal. Res.*, pp. 4–5, 2014, [Online]. Available: http://www.rds-london.nihr.ac.uk/How-to-design-a-study-find-funding/Health-economics/Cost-consequences-analysis.aspx.

[64] A. Curtis, L.A., Burns, “Unit Costs of Health & Social Care. 2020, University of Kent, Personal Social Services Research Unit.,” doi: 10.22024/UniKent%2F01.02.84818.

[65] Moran-Ellis, J., et al., Triangulation and integration: processes, claims and implications. Qualitative Research, 2006. 6(1): p. 45-59.

[66] Braun, V. and V. Clarke, *Using thematic analysis in psychology*. Qualatative Research in Psychology, 2006. 3(2): p. 77-101.

[67] Virginia Braun & Victoria Clarke(2019)Reflecting on reflexive thematic analysis, Qualitative Research in Sport, Exercise and Health,11:4,589-597,DOI: 10.1080/2159676X.2019.1628806

10 Appendix 1 – Logic model

<table>
<tr><th>Context</th><th>Individual staff barriers [TDF domain]</th><th>Intervention components [Behaviour change technique]: Individual staff-focused</th><th>Inputs</th><th>Outputs</th><th>Short-term Outcomes</th><th>Medium-/Long-term outcomes</th></tr>
<tr>
<td rowspan="3">Evidence suggests that care home staff vaccination reduces resident morbidity and mortality

The WHO recommends that at least 75% of staff should get vaccinated

Homes have a trusted relationship with the community pharmacy providing their residents' medication

Pharmacists are permitted to vaccinate staff in homes, but few do so due to the costs involved and demand uncertainty

Care staff employers have a responsibility to facilitate vaccination, but this is only of their many responsibilities</td>
<td>Environment; Behavioural Regulation
Lack time to go to GP or pharmacy to get vaccinated

Environmental context and resources
Some staff (e.g. agency) are ineligible for free vaccination.

Beliefs about consequences
Believe they are fit and healthy so do not need vaccination

Believe the vaccine is ineffective or causes flu

Social influences
Staff question why they should get vaccinated when others do not.</td>
<td>Restructuring the physical environment; Review goal.
Community pharmacies commissioned to proactively offer regular staff vaccination clinics in homes at convenient times. If uptake is low, line managers talk to staff to understand why

Restructuring the physical environment
NHS funded vaccination available for all staff.

Information about health consequences and others' approval; Salience of consequences; Framing/re-framing
Two short videos featuring: (1) Residents and vulnerable (older and younger) staff explaining that others' non-vaccination causes their flu and describing their experience of it. (2) Explanation of why vaccines cannot be 100% effective but still work and why it cannot cause flu.

Emphasising a message of protecting yourself and your own family. Integrated into existing staff processes and reinforced via posters.</td>
<td rowspan="3">Clinical Commissioning Group resources to commission pharmacies; provide incentives and monitoring services

Vaccine cost for staff not eligible for NHS vaccination

Pharmacist and dispenser time, PPE and other service delivery costs

Care home manager and staff time

Videos and information campaign resources</td>
<td rowspan="3">No. of pharmacy visits to homes

Length of pharmacy visit to home

No. of times videos played.

No. of posters displayed

No. of incentive payments made to homes</td>
<td rowspan="3">Increase in staff flu vaccination rates

Residents have fewer episodes of flu-like illness, GP visits and hospitalisations

Reduced resident mortality

Fewer staff sick days

Reduced staff costs and NHS costs

Fewer staff misconceptions around vaccination

Residents have the same carer more often

Staff better appreciate how their behaviour affects residents</td>
<td rowspan="3">Managers develop their own flu campaign

Better infection control and occupational health culture

Reduced health inequities

Higher quality old age care

Higher life expectancy

Improved mental and physical health

More financially sustainable homes

Staff more willing to take vaccines in general

Vaccination model adapted and used in other social care settings</td>
</tr>
<tr><th colspan="2">Organisation-level strategies</th></tr>
<tr><td colspan="2">Incentives
Care homes receive (CQUIN-like) incentive payment and certificates for achieving >70% of staff vaccinated

Monitoring and feedback
Regular monitoring of and feedback on vaccination uptake and efforts to promote</td></tr>
</table>

11 Appendix 2 – Study Within A Trial (SWAT)

Research Protocol

Title:	Better Targeting Behaviour Change Interventions Using Decision-Making Characteristics – a Study Within A trial (SWAT)
Co-Chief Investigator (and UEA Lead):	Dr Amrish Patel
Co-Applicant:	Prof Ted Turocy
	Dr Oana Borcan
Funder	British Academy/Leverhulme Small Research Grants
Award	£10,000
Funder Reference	SRG22\220793

Lay Summary

Solving social problems often involves “behaviour change”. Policymakers in areas from health to environment increasingly rely on behaviour change interventions to achieve their goals. While we know different people react differently to such interventions, not enough is known about how to target these interventions at those for whom they will work best. This project will assess the feasibility of using behavioural economics methods to elicit decision-making characteristics (e.g. preferences/beliefs) to better understand which people an intervention works on.

We will do this in the context of an intervention tackling a particularly topical problem, low influenza vaccination uptake amongst care home staff. A randomised controlled trial will evaluate the effectiveness of this intervention in 78 care homes. We will elicit decision-making characteristics of care home staff before and after the intervention in 58 of these care homes, enabling us to understand which types of people the intervention works for, informing how to better target the intervention.

Background

Changing behaviour is critical to overcoming many important social problems. Recent advances in behavioural science have led to policymakers moving from away from solely relying on traditional policy instruments (e.g. taxes and legislation) and towards designing and implementing so-called “behaviour change interventions” (Dai et al. 2021).

These interventions use insights from behavioural economics and cognate disciplines to influence target behaviours. They can range from the “nudge” of a simple text-message reminder (Thaler and Sunstein 2009) to a suite of policy changes, jointly facilitating behaviour change.

However, despite their widespread use, there are concerns over the limited size of behaviour change achievable when such interventions are applied at scale (Dellavigna and Linos 2022). One solution to make them more effective is to ensure interventions are targeted at individuals for whom they are most likely to work, such as using “personalised nudging” (Mills 2020)but thisis difficult becuase an individual’s decision-making characteristics (e.g. preferences and beliefs) which determine their behaviour are typically unobservable, so policymakers cannot know which types of people (in terms of decision making characteristics) an intervention is most likely to work for.

In this project, we aim to test whether Behavioural Economics methods for eliciting decision-making characteristics (e.g. risk-aversion or prosociality) can help understand what types of individuals a behaviour change intervention works best for, which may inform the use of these interventions in clinical trials. If so, policymakers can use existing evidence on how such characteristics relate to observable features of populations (e.g. older people are typically more risk-averse) to better target interventions when implemented at scale.

The proposed project will be nested within the FluCare Randomised Control Trial (ISRCTN22729870). Specifically, we will test whether decision-making characteristics of care home staff elicited using behavioural economics methods are predictive of the effectiveness of the FluCare intervention.

<u>Research Questions</u>

RQ 1: Does the effectiveness of the behaviour change intervention vary by staff member’s risk-preferences?

RQ 2: Does the effectiveness of the behaviour change intervention vary with staff member’s prosociality?

RQ 3: Does the effectiveness of the behaviour change intervention vary by a staff member’s beliefs about other’s vaccination behaviour?

We will also address an additional research question may which shed light on whether the intervention works by affecting any of the decision-characteristics under study:

RQ 4: Does the behaviour change intervention change staff’s risk-aversion, prosociality or beliefs about other’s vaccination behaviour?

<u>Population</u>

Inclusion criteria:

Care home staff working in care homes that are participating in the FluCare trial

Exclusion Criteria

Care home staff working in care homes that are participating in the FluCare trial, and which have been selected to take part in the embedded process evaluation

Recruitment

Care home managers are the recruitment facilitators for this study. The care home manager will be sent a care home specific invitation and link to distribute to their to staff using their normal communication system. This maybe via text, WhatsApp, Email or an alternative platform. The link will direct the staff member to the participant information sheet (PIS), consent form and baseline questionnaire to their staff.

Consent

Upon opening of the link to the study, Care home staff will be invited to read the participant information sheet and provide e-consent to participate in the study. Upon completion of the e-consent form, the participant will be emailed a copy of the PIS and their completed consent form. The participant can decide how long they wish to take between reading the PIS and consenting to participate in the study.

Outcome Measure

A decision choice questionnaire has been developed for this study. It includes:-

incentivised choice tasks developed to elicit our three decision-making characteristics of interest (i.e. risk-aversion, prosociality and beliefs about others' vaccine uptake). These types of incentivised choice tasks permit large samples of participants with attenuated biases given the use of real (incentivised) choices rather than hypothetical choices/statements. Choice data generated using this method is now standard in economics and predictive of many real world (large stakes) decisions (Charness and Fehr 2015).

The following methods will be used to elicit each of the preferences using an online survey:

- Risk-aversion: measured using the Holt and Laury (2002) lottery choice task. Respondents will be presented with a sequence of binary choices between a certain amount of money and a lottery. The amount of risk involved in the lottery will increase in each successive choice. The later in the sequence that a respondent switches from choosing the lottery to the certain amount, the more risk-averse the respondent is.
- Prosociality: measured via a charitable giving dictator game. Respondents will be given £7 and asked to split the money between themselves and a donation to Age Concern (a charity for older adults). We chose this charity because it reflects that care home staff are getting vaccinated to protect older individuals. In contrast to some existing studies which use a public good game to measure prosociality motivating vaccination, a dictator game is more

appropriate for care home staff becuase the vast majority of benefits (in terms of avoiding life-threatening illness) accrue to residents rather than staff.

- Beliefs about the vaccination of others will be measured using a standard incentivised belief-elicitation method, such as that by Krupka and Weber (2013). First respondents will be asked whether they intend to get vaccinated for flu. They will then be asked to guess what share of care home staff responding to the survey stated they will get vaccinated this year. Respondents will be paid more the closer their guess is to the true share of staff that stated that they will get vaccinated this year.

Participants will be asked to complete the questionnaire at baseline, before the start of the FluCare intervention, and approximately four months later. With participant consent, direct emails and text messages will be sent to participants requesting completion of the follow-up questionnaire. Care home managers will also be asked to remind their staff via their own communication channels that the follow-up questions are due to be completed. Reminders will also be sent, because these were found to be critical to increase responses to the FluCare Mechanism of Action Questionnaire survey.

Each respondent will receive a fixed £5 payment and a variable payment. The variable payment will be determined by their response to one of their choices in the survey (randomly selected). This is done to avoid income effects that can lead to respondents not engaging with the survey once they think believe they have earned enough.

Since this research is nested within the FluCare trial, we will be able to link our survey results to the FluCare database which will give us care home level data on aggregate flu vaccination status and additional data (e.g. number of sick days taken) and their workplace (e.g. aggregate resident illness in their care home).

Data Management

Data will be collected using an online O-TREE form developed and maintained by The Laboratory for Economic and Decision Research (LEDR), and hosted by the University of East Anglia. LEDR is a dedicated space used exclusively for economic experiments conducted by our academics and research students.

Sample Size

We aim to have a sample size of 550 for the baseline survey (275 per FluCare trial arm) and assume an 18% attrition rate (based on the FluCare's survey experience last flu season). For the follow-up survey we aim to have 450 participants (225 participants per trial arm) all of whom also answered the baseline survey. However, over recruitment in one arm compared to the other will be permitted. To maximise the likelihood of balanced responses between the FluCare trial arms, and also from a wide range of homes, reminders will be sent sequentially to staff within homes (and the trial arm) with many responses sent later.

The number of participants completing the questionnaire at baseline will be monitored against the budget spend. Since costs are calculated at a maximum participant payment available, if budget

permits and we have less attrition than expected, then the survey will be kept open until the budget is exhausted to improve statistical power.

Analysis

We will estimate OLS regressions where the dependent variable is vaccination status and the right-hand side variables of interest will be interactions between whether a staff member comes from a care home which received the intervention and each decision-making characteristic (i.e. risk aversion, prosociality or beliefs). A positive significant coefficient on such an interaction would mean that the intervention is more likely to work (i.e. lead to vaccination) on staff with a higher degree of the decision-making characteristic (e.g. risk-aversion).

Given how busy care home staff are, we anticipate significant selection amongst those staff who respond to our survey. However, we have considerable staff-level data on all employees of the 58 care homes in the FluCare trial dataset (approximately 2900 staff in 58 care homes, each with approximately 50 staff). We will examine test whether respondents differ significantly from the population of the care home staff in the trial based on observable characteristics (e.g. sociodemographics, number of sick days etc) and use existing literature to inform whether any biases are implied by the selection observed.

A data analysis plan will be written and published prior to data analysis.

Dissemination

Findings published in the literature on the determinants of vaccine uptake, but also the broader literature on understanding the effectiveness of behaviour change interventions.
